# Odor impact of volatiles emitted from marijuana, cocaine, heroin and their surrogate scents

**DOI:** 10.1016/j.dib.2015.09.053

**Published:** 2015-10-23

**Authors:** Somchai Rice, Jacek A. Koziel

**Affiliations:** a Department of Agricultural and Biosystems Engineering, Iowa State University, Ames, IA 50011, USA; b Interdepartmental Toxicology Graduate Program, Iowa State University, Ames, IA 50011, USA

## Abstract

Volatile compounds emitted into headspace from illicit street drugs have been identified, but until now odor impact of these compounds have not been reported. Data in support of identification of these compounds and their odor impact to human nose are presented. In addition, data is reported on odor detection thresholds for canines highlighting differences with human ODTs and needs to address gaps in knowledge. New data presented here include: (1) compound identification, (2) gas chromatography (GC) column retention times, (3) mass spectral data, (4) odor descriptors from 2 databases, (5) human odor detection thresholds from 2 databases, (6) calculated odor activity values, and (7) subsequent ranking of compounds by concentration and ranking of compounds by odor impact (reported as calculated odor activity values). For further interpretation and discussion, see Rice and Koziel [1] and Rice [2].

**Specifications table**

**Table:** 

Subject area	*Chemistry*

More specific subject area	*Forensics, Analytical Chemistry, Olfactometry*
Type of data	*Table*
How data was acquired	*Multidimensional gas chromatography (Agilent 6890), mass spectroscopy (Agilent 5973), olfactometry (MOCON, Round Rock, TX).*
Data format	*Analyzed mass spec using Automatic Mass Spectral Deconvolution and Identification System (AMDIS) (NIST, Gaithersburg, MD)*
Experimental factors	*Volatiles emitted from marijuana, cocaine, and heroin samples were collected on Carboxen/polydimethylsiloxane (PDMS) solid-phase microextraction (SPME) fiber at room temperature, static, for 1 h.*
Experimental features	*SPME fibers were thermally desorbed in a multidimensional gas chromatography-mass spectrometry-olfactometry (MDGC-MS-O) instrument, allowing for simultaneous chemical and sensory analysis. Surrogate scents for each drug were also analyzed as previously stated, and aromas were compared using calculated odor activity values (OAVs)*.
Data source location	*Department of Agricultural and Biosystems Engineering at Iowa State University, Ames, IA 50011*
Data accessibility	*Data is with this article.*

**Value of the data**

• This data is the most comprehensive summary of volatiles emitted from real and surrogate scents of marijuana (221, 78), cocaine (153, 15), and heroin (41, 19), respectively, to date.
• This data includes organoleptic percepts from 2 known databases, odor detection thresholds from 2 benchmark databases, significant ions from mass spectral data, and calculated odor activity values (OAVs, if available) for each compound.
• This data shows rank of drug volatiles by concentration in relation to the rank by odor impact (as calculated OAV).
• Odor activity value data can open up new ways of forensic drug analysis.
• Data from previous research on canine odor detection thresholds (ODTs) is reported for further insight, highlighting differences with human ODTs and needs to address gaps in knowledge.

## 1 Data

### 1.1 CAS Registry Number

A CAS Registry Number is a unique numeric identifier that corresponds to only one substance. The CAS has no chemical significance, but can be used as a link to more information about a specific chemical substance [3]. CAS number is useful to identify a compound that has multiple synonyms. CAS numbers were used in all data tables in this report.

### 1.2 Odor Detection Threshold (ODTs)

Published ODT values are not fixed numbers, but are set to represent the *lowest* concentration that 50% of the population can detect [4]. For the purposes of calculating odor activity values, standardized human ODTs from Devos et al. [5] were used when available. The compilation from Devos et al. contained a total of 2075 ODT values in air for 641 chemical compounds, gathered from 372 references. ODTs were weighted and averaged [5]. If ODT for a compound was not given in Devos et al., the LRI Database [6] was used. LRI database contains 1500+ records on ODT and odor percepts [6]. ODTs for canines were compiled from previous literature. See Rice and Koziel [1] for full discussion on human and canine ODTs. ODTs were used in reporting data in Table 1, Table 2, Table 3, Table 4, Table 5.

### 1.3 Column retention time in chromatography

Column retention time (RT, min) is the time between sample introduction via thermal desorption in the gas chromatography (GC) inlet and the analyte peak reaching the mass spectrometer and/or sniff port at the end of the analytical column. It was not appropriate to use retention indexes (Kovats RI) for identification because: (1) the non-polar and polar columns were connected in series when analyzing in multidimensional GC; (2) use of indices of medium polarity column could lead to large errors for compounds that are affected by one of the columns more than the other. Known retention times of standards previously analyzed on this system were used for compound identification, and indicated by + symbol in Table 3, Table 4, Table 5. RTs are also reported in Table 3, Table 4, Table 5.

### 1.4 Aroma descriptors

Aroma descriptors were compiled from Flavornet and The Good Scents Company. Flavornet has aroma descriptors from 738 compounds, compiled from studies using GC-olfactometry [7]. The Good Scents Company is dedicated to providing organoleptic information to the flavor, food and fragrance industry [8]. Aroma descriptors from these 2 databases were used in reporting data in Table 3, Table 4, Table 5.

### 1.5 Sample code

Aromas were characterized by human nose from volatiles emitted into the headspace of illicit marijuana, cocaine, and heroin. Various states of seizure were examined: (1) 50 kg of marijuana in a cloth military style duffel bag (**Sample Code A1–A3**); (2) 1 g marijuana packaged in a plastic zip-top sandwich bag (**Sample Code A4–A5**); (3) 1 g old, desiccated marijuana with no packaging (**Sample Code A6–A7**); (4) plastic zip-top sandwich bag with 1 g marijuana removed (**Sample Code B1–B4**); (5) 1 g crack cocaine packaged as tear drops (**Sample Code D1**); (6) 1 g cocaine adulterated with Levamisole (**Sample Code D2**); (7) 1 kg evidence pack containing cocaine (**Sample Code D3**); (8) 1 g cocaine in an opened plastic bag (**Sample Code D4–D5**); (9) 1 g heroin seized in 1997 (**Sample Code F1**); (10) 1 g heroin seized in 2010 (**Sample Code F2**). Sigma Pseudo™ Narcotic Scent Marijuana formulation (Fluka, #P7309) (**Sample Code C1–C3**), Sigma Pseudo™ Narcotic Scent Cocaine formulation (Fluka, #P2423) (**Sample Code E1**), and Sigma Pseudo™ Narcotic Scent Heroin formulation (Fluka, #P2548) (**Sample Code G1**) were purchased from Sigma-Aldrich (St. Louis, MO). These sample codes were used in reporting data in Table 3, Table 4, Table 5.

### 1.6 Target mass spec libraries, models, and net % match, peak area counts

AMDIS (NIST, Gaithersburg, MD) software was used for identification of unknown compounds. Six specialty mass spectral libraries were used for compound identification: NISTEPA (1086 compounds in the EPA’s ‘list of lists’), NISTDRUG (739 compounds in the Canadian AAFS Toxicology Section MS Database Committee and the Association of Official Racing Chemists libraries), NISTFF (991 compounds in the Philip Morris Flavor and fragrance collection), NISTTOX (1213 compounds represented in Finnigan Corporation’s Toxicology library), NISTFDA (415 compounds in an FDA collection of mass spectra), and NISTCW (62 compounds relevant to detection of chemical weapons).

A model is the mass-to-charge ratio (*m/z*) of a deconvoluted peak, and are listed in order of highest to lowest relative abundance. For example, under a ‘Models’ column heading, 2: 58 88 signifies 2 models with *m/z* 58 and 88 were used for identification.

Net % Match is the final match quality value (100=perfect match) between the deconvoluted component and the target library spectra. The minimum match value was set at 65 for all analysis of this data.

Peak area counts (PAC) refers to the relative abundance of the analyte, or the area under the chromatographic peak. The mass detector was assumed to have equal response factors for each compound, for the purposes of calculating OAV.

These parameters were used in reporting data in Table 3, Table 4, Table 5.

### 1.7 Odor activity values (OAV)

OAV is defined as the unit less ratio of concentration of a compound in gas phase to the odor detection threshold. For illustrative purposes, the PAC was used for the concentration value of each compound. See Rice and Koziel [1] and Rice [2] for further discussion on OAV. This ratio was used to calculate the OAVs reported in Table 3, Table 4, Table 5.

### 1.8 Ranking definitions

Compounds from each drug were ranked by concentration (highest concentrated=ranked #1) and then by calculated OAV (highest odor impact=ranked #1). In most cases, there was no apparent correlation between chemical concentration and odor impact, i.e., rank #1 by concentration did not usually rank as 1 by OAV. This ranking and sorting was used to report data in Table 6, Table 7, Table 8.

## 2 Experimental design, materials and methods

### 2.1 Surrogate scent formulations

Sigma Pseudo™ Narcotic Scent Marijuana formulation composition is listed as pyrogenic colloidal silica (1%), cellulose (98.5%), butane-2,3-diol (0.4%), and p-mentha-1,4-diene (0.1%). Sigma Pseudo™ Narcotic Scent Cocaine formulation composition is listed as cellulose (98.9%), pyrogenic colloidal silica (1%), and methyl benzoate (0.1%). Sigma Pseudo™ Narcotic Scent Heroin formulation composition is listed as cellulose (74.1%), o-acetylsalicylic acid (25.2%), acetic acid (0.3%), and pyrogenic colloidal silica (0.3%).

### 2.2 Methodology

Carboxen/PDMS, 85 μm Stable-flex, 24 gauge SPME fibers were used (Sigma-Aldrich, St. Louis, MO, USA). Briefly, experimental conditions were as follows: drugs were placed in separate, pre-cleaned and oven-baked 16 ounce mason jars with modified lids. The Carboxen/PDMS SPME fibers were exposed to the headspace and volatiles were passively extracted; equilibration time was the same as extraction time (1 h at ambient temperature). When the extraction step was completed, the SPME fiber was retracted, wrapped in pre-baked aluminum foil, placed in a pre-cleaned mason jar, and transported back to the laboratory in a cooler on ice. In the laboratory, fibers were stored as described above in a 4 °C refrigerator pending placement into the heated injection port of the MDGC-MS-O for thermal desorption and analysis.

MDGC-MS-O analysis was performed on an Agilent 6890 GC, with a restrictor guard column, non-polar capillary column (BP-5, 56 m × 530 μm inner diameter × 1.00 μm thickness, SGE, Austin, TX, USA) and polar capillary column (BP-20, 25 m × 530 μm inner diameter × 1.00 μm thickness, SGE, Austin, TX, USA) connected in series. Outflow from analytical column was held at 7.0 cc/min. Sample flow was split 3:1 via open split interface to the sniff port and mass spectrometer, respectively, as determined by restrictor column inner diameter. Desorption time was 2 min in splitless mode at 270 °C under flow of helium carrier gas (99.995% purity). Analysis of the same fiber immediately after sample injection, revealed no carry over, with all compounds desorbed in the initial analysis. The oven temperature was programmed as follows: 40 °C for 3.00 min, then increased to 220 °C at a rate of 7.00 °C per min, and held for 11.29 min (40 min total run time). The carrier gas was set at constant pressure at the midpoint (junction point of the non-polar and polar column) at 5.8 psi. Transfer line to the MS was set at 240 °C; transfer line to the sniff port was set at 240 °C with humidified air set at 8.00 psi. MS heated zones were 150 °C for the quadrupole and 230 °C for the source. Mass spectrometer parameters were electron impact (EI), electron energy set to 70 eV, with acquisition range m/z 33–280.

The instrument was tuned daily and analysis of column blanks did not show any contaminating compounds. Analysis of blank trip fiber (an unloaded SPME fiber taken to the site and back, stored with fibers to be analyzed) at the end of each sampling run did not demonstrate contaminating compounds. VOCs were identified tentatively using the Automatic Mass Spectral Deconvolution and Identification System (AMDIS) (National Institute of Standards and Technology, Gaithersburg, MD) and six specialty mass spectral libraries provided derived from the NIST05/EPA/NIH mass spectral database. Known retention times of standards previously analyzed on this system were used for identification. Chemical standards available in house were analyzed to match retention times and mass spectra of unknown compounds. Select reference standards were used for identification, purchased from Sigma-Aldrich (St. Louis, MO, USA). These standards are indicated with ‘+’ in Table 3, Table 4, Table 5. Each sample (as outlined in Section 1.5) was collected on a single SPME fiber, each fiber sample was analyzed by one panelist. The same panelist analyzed all samples with volatiles from each drug and surrogate scent formulation.

## Figures and Tables

**Table 1:** Comparison of odor detection thresholds and odor activity values between canines (based on Passe and Walker [9]) and humans (based on Devos et al. [5]).

**Source reference in**[9]	**Methods**	**Compound**	**CAS**	**Canine ODT**[9]**(ppm)**	**Human ODT**[5]**(ppm)**	**ODT**_**C**_**:ODT**_**H**_	**Canine OAV of 1 ppm**	**Human OAV of 1 ppm**	**OAV**_**C**_**:OAV**_**H**_
Neuhaus [10]	Dogs chose from 3 ***odor ports***. Pushing a box behind the correct port uncovered sugar for reward.	Acetic acid	64-19-7	4.99E-11	1.45E-01	3.44E-10	2.00E+10	6.90E+00	2.90E+09
Propanoic acid	79-09-4	3.09E-11	3.55E-02	8.70E-10	3.24E+10	2.82E+01	1.15E+09
Butyric acid	107-92-6	1.46E-12	3.89E-03	3.76E-10	6.84E+11	2.57E+02	2.66E+09
Pentanoic acid	109-52-4	5.36E-12	4.79E-03	1.12E-09	1.87E+11	2.09E+02	8.94E+08
Hexanoic acid	142-62-1	7.67E-12	1.26E-02	6.09E-10	1.30E+11	7.94E+01	1.64E+09
Octanoic acid	124-07-2	1.20E-11	3.98E-03	3.01E-09	8.34E+10	2.51E+02	3.32E+08
									
Ashton, Eayrs and Moulton [11]	Crucibles containing odorous solutions was placed on the floor. Dog alerted by sitting when odor was present.	Formic acid	64-18-6	1.30E+03	2.82E+01	4.60E+01	7.71E-04	3.55E-02	2.17E-02
Acetic acid	64-19-7	1.73E+02	1.45E-01	1.19E+03	5.77E-03	6.90E+00	8.37E-04
Propanoic acid	79-09-4	1.78E+01	3.55E-02	5.01E+02	5.63E-02	2.82E+01	2.00E-03
Butyric acid	107-92-6	3.67E+00	3.89E-03	9.44E+02	2.72E-01	2.57E+02	1.06E-03
Pentanoic acid	109-52-4	5.24E+01	4.79E-03	1.09E+04	1.91E-02	2.09E+02	9.14E-05
Hexanoic acid	142-62-1	3.20E+01	1.26E-02	2.54E+03	3.13E-02	7.94E+01	3.94E-04
Heptanoic acid	111-14-8	1.76E+01	2.75E-02	6.39E+02	5.69E-02	3.64E+01	1.57E-03
Octanoic acid	124-07-2	8.11E+00	3.98E-03	2.04E+03	1.23E-01	2.51E+02	4.91E-04
									
Moulton, Ashton, and Eayrs [12]	Crucibles containing odorous solutions was placed on the floor. Dog alerted by sitting when odor was present.	Formic acid	64-18-6	1.96E-02	2.82E+01	6.96E-04	5.09E+01	3.55E-02	1.44E+03
Acetic acid	64-19-7	5.73E-04	1.45E-01	3.95E-03	1.74E+03	6.90E+00	2.53E+02
Propanoic acid	79-09-4	1.23E-05	3.55E-02	3.46E-04	8.13E+04	2.82E+01	2.89E+03
Butyric acid	107-92-6	4.95E-07	3.89E-03	1.27E-04	2.02E+06	2.57E+02	7.85E+03
Pentanoic acid	109-52-4	1.55E-05	4.79E-03	3.23E-03	6.47E+04	2.09E+02	3.10E+02
Hexanoic acid	142-62-1	3.13E-06	1.26E-02	2.48E-04	3.20E+05	7.94E+01	4.03E+03
Heptanoic acid	111-14-8	5.55E-07	2.75E-02	2.02E-05	1.80E+06	3.64E+01	4.95E+04
Octanoic acid	124-07-2	1.12E-07	3.98E-03	2.81E-05	8.93E+06	2.51E+02	3.56E+04
Isobutyric acid	79-31-2	5.56E-07	1.95E-02	2.85E-05	1.80E+06	5.13E+01	3.51E+04
									
Moulton and Marshal [13]	Trial was initiated by manipulating a treadle, dogs chose from 3 ***odor ports***. Alert was placing nose in correct odorant for 5 seconds.	α-ionone	127-41-3	4.02E-13	5.75E-05	6.99E-09	2.49E+12	1.74E+04	1.43E+08
									
Marshall, Blumer and Moulton [14]	Same test apparatus as Moulton and Marshal (1976). 1 sample port, alert was keeping nose in port for 5 sec.	Pentanoic acid	109-52-4	1.51E-07	4.79E-03	3.15E-05	6.62E+06	2.09E+02	3.17E+04
									
Krestel, Passe, Smith and Jonsson [15]	Conditioned suppression using ***odor ports.***	Amyl acetate	628-63-7	1.93E-07	3.09E-02	6.23E-06	5.19E+06	3.24E+01	1.60E+05

REF=reference; ODT=odor detection threshold; OAV=odor activity value; ODT_C_=canine odor detection threshold; ODT_H_=human odor detection threshold; OAV_C_=odor activity value for canines; OAV_H_=odor activity value for humans. All gas phase calculations assumed 1 atm at 25 °C.

**Table 2:** Comparison of ODT and OAV in canines vs. humans in two recent field studies.

**REF**	**Method**	**Mixture ratio**	**Compound**	**CAS**	**Conc. tested (ppm)**	**% of canines alerted**	**Canine ODT (ppm)**	**Human ODT (ppm)**	**ODT**_**C**_**:ODT**_**H**_	**Canine OAV of 1 ppm**	**Human OAV of 1 ppm**	**OAV**_**C**_**:OAV**_**H**_
Lorenzo, Wan, Harper, Hsu, Chow, Rose, Furton [16]	Scent solution was spiked onto filter paper, placed in a metal box with holes drilled on top.		Insosafrole	120-58-1	6.76E+02	0						
	Phorone	504-20-1	6.27E+02	4	6.27E+02			1.60E-03		
	Camphor	76-22-2	6.43E+02	0		5.13E-02			1.95E+01	
	Piperonal	120-57-0	6.52E+02	17	6.52E+02	4.79E-03	1.36E+05	1.53E-03	2.09E+02	7.35E-06
	Safrol	94-59-7	6.61E+02	0		9.55E-03			1.05E+02	
	Benzaldehyde	100-52-7	9.63E+02	9	9.63E+02	4.17E-02	2.31E+04	1.04E-03	2.40E+01	4.33E-05
	Acetic acid	64-19-7	1.71E+03	0		1.45E-01			6.90E+00	
	1-phenyl-2-propanol	100-86-7	6.35E+02	9	6.35E+02			1.58E-03		
	Acetophenone	98-86-2	8.37E+02	0		3.63E-01			2.75E+00	
1:1	MD-P2P	4676-39-5	5.49E+01	0						
	Piperonal	120-57-0	6.52E+01	0		4.79E-03			2.09E+02	
3:1	MD-P2P	4676-39-5	1.65E+02	0						
	Piperonal	120-57-0	6.52E+01	0		4.79E-03			2.09E+02	
5:1	MD-P2P	4676-39-5	2.75E+02	0						
	Piperonal	120-57-0	6.52E+01	0		4.79E-03			2.09E+02	
10:1	MD-P2P	4676-39-5	5.49E+02	0						
	Piperonal	120-57-0	6.52E+01	0		4.79E-03			2.09E+02	
5:1	MD-P2P	4676-39-5	1.37E+03	0						
	Piperonal	120-57-0	6.52E+01	0		4.79E-03			2.09E+02	
	MDMA	NA	5.07E+00	0						
	MD-P2P	4676-39-5	5.49E+02	0						
	Piperonal	120-57-0	6.52E+03	83		4.79E-03			2.09E+02	
	Methamphetamine Pharm Grade	537-46-2	3.28E+05	0						
Unknown mixture	Methamphetamine Street Sample	NA		100						
												
Williams and Johnston [17]	Cotton balls were spiked with target odor and placed in a can^Δ^.		Allyl sulfide	592-88-1	6.01E+05	>80%	6.01E+05			1.66E-06		
	Cumene	98-82-8	5.68E+05	>80%	5.68E+05	2.40E-02	2.37E+07	1.76E-06	4.17E+01	4.22E-08
	dimethylthiazole	541-58-2	7.39E+05	>80%	7.39E+05			1.35E-06		
	α-pinene	80-56-8	4.99E+05	>80%	4.99E+05	6.92E-01	7.21E+05	2.00E-06	1.45E+00	1.39E-06
	benzaldehyde	100-52-7	7.80E+05	>80%	7.80E+05	3.00E-03	2.60E+08	1.28E-06	3.33E+02	3.85E-09
	Menthol	89-78-1	4.51E+05	>80%	4.51E+05	4.17E-02	1.08E+07	2.22E-06	2.40E+01	9.24E-08
	Cyclohexanone	108-91-1	7.65E+05	>80%	7.65E+05	7.08E-01	1.08E+06	1.31E-06	1.41E+00	9.25E-07
	Eucalyptol	470-82-6	4.74E+05	>80%	4.74E+05	1.62E-02	2.93E+07	2.11E-06	6.17E+01	3.42E-08
	Pentanethiol	110-66-7	6.39E+05	>80%	6.39E+05	1.20E-04	5.31E+09	1.57E-06	8.32E+03	1.88E-10
	Toluene	108-88-3	7.48E+05	>80%	7.48E+05	1.55E+00	4.83E+05	1.34E-06	6.45E-01	2.07E-06

REF=reference; MD-P2P=3,4-methylenedioxyphenyl-2-propanone; Mixture ratio=ratio of MD-P2P to Piperonal; ODT=odor detection threshold; OAV=odor activity value; ODTc=canine odor detection threshold; ODT_H_=human odor detection threshold; OAVc=odor activity value for canines; OAV_H_=odor activity value for humans. ^Δ^Volume of can was not specified. In this table the can dimensions were assumed to be a cylinder of radius 5.08 cm, 4.62 cm height, displaced volume of cotton balls was not accounted for. All gas phase calculations assumed 1 atm at 25 °C.

**Table 3:** Summary of VOCs emitted from all illicit marijuana samples (sample code A and B in Section 1.5) and Sigma Pseudo™ Narcotic Scent Marijuana formulation (sample code C in Section 1.5) and sampled over 1 h at room temperature. Sigma Pseudo™ Narcotic Scent Marijuana formulation is indicated by underlined fonts.

**Compound**	**CAS**	**RT (min)**	**Published descriptors**		**Published ODT (ppm)**		**Sample code**			**Models**	**Net % match**	**PAC**	**OAV**
			**Flavornet**[7]	**TGSC**[8]	**LRI and Odor**[6]	**Devos et al**. [5]							
Ethylene oxide	**75-21-8**	**1.07**				**8.51E+02**	A	1		2: 44 45	66	1.51E+06	1.77E+03
A	3			66	2.12E+06	2.49E+03
A	4			65	3.37E+06	3.96E+03
A	7		2: 43 42	89	8.62E+03	1.01E+01
B	1		2: 44 43	66	3.75E+06	4.40E+03
B	4		4: 44 45 129 43	66	1.86E+06	2.18E+03
C	1		3: 44 45 46	66	1.35E+06	1.59E+03
C	2		2: 44 43	85	2.14E+05	2.51E+02
C	3		4: 44 46 43 131	67	1.36E+06	1.60E+03
+2-nitropropane	79-46-9	1.13				7.24E+00	A	5		2: 41 43	75	6.30E+03	8.69E+02
A	6		4: 43 39 56 42	83	4.16E+04	5.74E+03
2,4-dimethylpentane	108-08-7	1.20				8.71E+01	A	7		2: 57 43	66	8.15E+03	9.36E+01
Isobutane	75-28-5	1.22				1.00E+01	A	1			84	2.20E+07	2.20E+06
A	2		13: 43 41 57 72 39 55 56 38 40 73 62 66 65	84	2.02E+07	2.02E+06
A	3		11: 43 42 41 57 72 40 53 51 38 73 63	85	1.47E+07	1.47E+06
A	4		5: 57 42 43 41 39	67	2.03E+04	2.03E+03
A	5		10: 43 42 41 39 72 55 50 73 71 58	84	7.18E+06	7.18E+05
A	6		4: 43 39 56 42	88	4.16E+04	4.16E+03
A	7		10: 43 42 41 57 39 72 55 56 73 37	85	2.94E+06	2.94E+05
B	1		14: 43 42 41 57 72 39 56 55 71 50 70 53 38 37	85	2.20E+06	2.20E+05
B	2		7: 42 41 72 53 55 56 38	84	7.00E+05	7.00E+04
B	3		4: 43 42 41 39	88	2.49E+04	2.49E+03
B	4		4: 42 43 57 72	81	6.45E+04	6.45E+03
+Acetaldehyde	75-07-0	1.27	Pungent, Ether	Pungent, Ethereal, Aldehydic, Fruity	1.50E-02	1.86E-01	A	4		2: 44 42	91	3.10E+04	1.67E+05
A	6		2: 43 44	90	2.69E+04	1.44E+05
A	7		2: 43 42	88	8.62E+03	4.63E+04
B	2			89	6.11E+03	3.28E+04
B	3			96	2.85E+04	1.53E+05
B	4		2: 44 43	96	8.88E+04	4.77E+05
C	2		2: 43 44	95	2.95E+04	1.58E+05
C	3		2: 43 41	96	6.95E+04	3.73E+05
Trichloromonofluoromethane	75-69-4	1.27					B	1		2: 103 101	75	4.34E+03	
B	4		2: 101 103	81	1.72E+04	
2,3-dimethylbutane	79-29-8	1.28					A	6		3: 43 71 42	73	1.06E+04	
B	2			66	5.01E+03	
B	3		4: 43 42 41 39	71	2.49E+04	
Ethylenimine	151-56-4	1.30					A	5		1: 41	70	5.22E+04	
A	6		3: 43 42 39	81	2.30E+04	
B	2			81	5.01E+03	
B	3		4: 43 42 41 39	83	2.49E+04	
+Ethyl ether	60-29-7	1.31		Ethereal			B	2		1: 59	86	2.37E+04	
Ketene	463-51-4	1.31					A	4			80	3.54E+03	
A	7		3: 41 42 59	74	3.81E+04	
B	1		3: 42 41 55	72	1.04E+05	
C	2		2: 41 42	73	3.11E+03	
Isoprene	78-79-5	1.33					A	4		3: 39 53 51	85	3.34E+04	
A	5		1: 67	71	1.73E+04	
A	7			93	3.12E+04	
B	1		3: 67 53 65	69	2.08E+04	
B	3			77	4.59E+03	
B	4		5: 67 51 41 53 66	95	7.61E+04	
C	3		3: 67 39 53	81	1.42E+04	
(E)-1,3-Pentadiene	2004-70-8	1.34					B	4			94	2.13E+04	
+1,3-Pentadiene	504-60-9	1.34					B	4			94	2.13E+04	
Hexane	110-54-3	1.34	Alkane			2.19E+01	A	1		7: 41 76 57 56 86 43 39	69	1.33E+05	6.09E+03
A	2		4: 62 56 42 86	66	2.53E+04	1.16E+03
A	3		2: 56 41	88	8.55E+04	3.91E+03
A	4		5: 57 42 43 41 39	78	2.03E+04	9.27E+02
A	5		2: 41 57	75	1.84E+04	8.43E+02
A	6		4: 76 42 56 43	74	1.57E+05	7.18E+03
A	7		1: 86	86	5.53E+04	2.53E+03
B	1		2: 57 56	79	3.71E+04	1.69E+03
B	2		2: 43 57	74	3.37E+04	1.54E+03
B	3			67	2.96E+04	1.35E+03
B	4			81	1.82E+04	8.32E+02
4-methyldecane	2847-72-5	1.39					A	1		12: 43 42 71 41 57 39 70 55 56 86 38 69	66	2.55E+06	
A	2		13: 43 71 42 41 57 70 39 56 86 85 62 54 63	66	2.66E+06	
A	3		17: 43 42 41 70 86 56 50 40 57 38 65 63 51 69 37 85 67	65	4.43E+06	
A	4			75	1.66E+04	
A	5		10: 39 57 55 41 86 53 69 38 52 67	66	1.35E+06	
A	7			65	6.20E+05	
B	1		10: 43 42 41 56 57 39 85 86 69 54	65	8.52E+05	
2-methylpentane	107-83-5	1.39					A	1		12: 43 42 71 41 57 39 70 55 56 86 38 69	98	2.55E+06	
A	2		13: 43 71 42 41 57 70 39 56 86 85 62 54 63	98	2.66E+06	
A	3		3: 67 87 85	98	4.89E+06	
A	4		2: 43 41	80	1.89E+04	
A	7		12: 42 41 55 39 69 72 70 86 56 40 65 50	97	6.18E+05	
B	1		10: 43 42 41 56 57 39 85 86 69 54	98	8.52E+05	
B	2		6: 43 42 41 70 57 86	96	2.39E+05	
B	4		4: 43 57 71 70	85	3.50E+04	
3,4,5-trimethyl-1-hexene	56728-10-0	1.39					A	1		12: 43 42 71 41 57 39 70 55 56 86 38 69	68	2.55E+06	
A	2		13: 43 71 42 41 57 70 39 56 86 85 62 54 63	68	2.66E+06	
A	3		3: 67 87 85	68	4.89E+06	
A	5		8: 43 71 42 41 57 50 56 86	68	1.54E+06	
A	7			67	6.20E+05	
B	1		1: 70	68	2.04E+05	
C	1		7: 85 99 71 110 98 68 39	67	2.17E+05	
+γ-butyrolactone	96-48-0	1.40	Caramel, Sweet	Creamy, Oily, Fatty, Caramel			A	7		12: 42 41 55 39 69 72 70 86 56 40 65 50	71	4.33E+05	
Acrylic acid	79-10-7	1.40			2.95E-01	2.95E-01	B	1		3: 72 55 58	65	2.17E+04	7.36E+04
2,3,4-trimethylpentane	565-75-3	1.40					A	4			75	1.52E+04	
A	7		9: 43 70 41 55 57 53 56 54 50	77	2.03E+05	
3-methylpentane	96-14-0	1.45					A	1		5: 57 56 41 58 71	98	4.98E+05	
A	2		8: 57 56 41 71 39 58 54 85	99	5.18E+05	
A	3			98	6.94E+05	
A	4			87	2.06E+05	
A	6		2: 57 39	70	3.72E+04	
A	7			97	1.22E+05	
B	1		6: 57 41 56 58 55 51	95	2.92E+05	
B	2		1: 57	86	6.05E+04	
B	4		2: 57 56	91	5.56E+04	
2-methylaziridine	75-55-8	1.45					A	1		5: 57 56 41 58 71	86	4.98E+05	
A	2		8: 57 56 41 71 39 58 54 85	80	5.18E+05	
A	3			81	6.94E+05	
A	4		3: 56 41 57	77	1.54E+04	
A	5		5: 57 56 55 58 86	81	4.24E+05	
A	7		5: 57 56 41 53 39	81	1.67E+05	
B	1		6: 57 41 56 58 55 51	80	2.28E+05	
B	2		1: 57	78	6.05E+04	
B	4		2: 57 56	80	5.56E+04	
Isocyanatomethane	624-83-9	1.46					A	3			80	1.20E+04	
A	4			81	5.01E+04	
A	6		2: 57 39	80	1.03E+05	
A	7			85	1.22E+05	
B	1		2: 57 56	79	7.68E+03	
B	2		2: 56 57	79	7.77E+03	
B	4		2: 56 57	78	1.20E+03	
Cyanogen chloride	506-77-4	1.47					B	2		8: 61 63 62 97 100 35 47 37	74	1.69E+05	
1,2-dichloro-, (Z)-ethene	156-59-2	1.47				1.91E+01	B	1		4: 60 62 55 86	100	5.02E+05	2.63E+04
B	2		6: 96 98 59 62 60 47	91	1.71E+05	8.96E+03
+Furan	110-00-9	1.47		Ethereal	4.50E+03		B	2		2: 39 68	80	9.70E+03	2.16E+00
B	4		1: 68	68	4.04E+04	8.99E+00
1,1-dichloro ethene	75-35-4	1.47			3.55E+01		B	2			99	3.57E+05	1.01E+04
+Dimethylsulfide	75-18-3	1.51	Cabbage, Sulfur, Gasoline	Sulfury, Onion, Sweet corn, Vegetable, Cabbage, Tomato, Green, Radish		2.24E-03	A	2		3: 47 39 35	66	5.52E+04	2.47E+07
A	7		5: 46 45 47 61 35	94	9.43E+04	4.21E+07
Carbon disulfide	75-15-0	1.52		Sulfur, Cabbage, Vegetable		9.55E-02	A	4		4: 76 39 86 59	82	6.35E+04	6.65E+05
A	5		2: 44 39	84	7.99E+04	8.36E+05
+3-pentanone	96-22-0	1.53	Ether	Ethereal, Acetone		3.16E-01	A	4		2: 57 86	74	9.06E+04	2.86E+05
A	6			67	3.85E+04	1.22E+05
B	3		2: 57 86	66	2.69E+04	8.50E+04
+Butane	106-97-8	1.57				2.04E+02	A	1			79	3.90E+05	1.91E+03
A	2		2: 43 58	78	5.36E+05	2.62E+03
A	4		2: 43 42	83	1.85E+04	9.04E+01
A	6			83	2.61E+06	1.28E+04
A	7		5: 41 59 44 37 60	84	1.88E+06	9.21E+03
B	1		7: 43 58 42 39 37 44 60	83	2.99E+06	1.47E+04
B	2			77	1.35E+05	6.62E+02
B	3		6: 43 58 42 39 38 36	84	1.96E+06	9.59E+03
B	4			87	4.62E+04	2.26E+02
C	1			69	4.25E+04	2.08E+02
C	2		2: 43 42	67	6.65E+04	3.26E+02
C	3		3: 43 58 42	68	7.06E+04	3.46E+02
Hordenine	539-15-1	1.57					A	6		1: 58	66	3.41E+04	
+Propanal	123-38-6	1.59	Solvent, Pungent	Earthy, Alcohol, Wine, Whiskey, Cocoa, Nutty	1.00E-02	2.69E-02	A	1		2: 58 42	76	9.76E+04	3.63E+06
A	6			76	1.57E+04	5.85E+05
A	7			76	3.30E+04	1.23E+06
B	1		2: 57 58	75	7.59E+04	2.82E+06
B	2		1: 58	73	4.63E+04	1.72E+06
B	3		2: 58 57	77	5.57E+04	2.07E+06
B	4		1: 58	83	1.04E+05	3.88E+06
1-Propanamine, 3-dibenzo[b,e]thiepin-11(6H)-ylidene-N,N-dimethyl-, S-oxide	1447-71-8	1.61					A	1		2: 58 42	75	1.50E+05	
A	2		1: 58	73	7.42E+04	
A	6		5: 58 38 59 52 36	71	3.95E+05	
A	7			65	3.33E+04	
B	1		2: 57 58	68	7.59E+04	
B	2		1: 58	70	4.63E+04	
B	3			71	1.40E+04	
B	4		1: 58	68	1.24E+05	
+Acetone	67-64-1	1.66		Solvent		1.45E+01	A	1		4: 43 58 42 37	97	5.84E+05	4.04E+04
A	2		2: 43 58	96	5.36E+05	3.71E+04
A	3		2: 43 58	81	4.96E+04	3.43E+03
A	4			98	8.97E+05	6.20E+04
A	6		7: 43 58 42 39 57 38 44	99	2.71E+06	1.88E+05
A	7		10: 43 58 42 39 41 38 37 44 36 59	99	4.98E+06	3.45E+05
B	1		7: 43 58 42 39 37 44 60	99	2.99E+06	2.07E+05
B	2			93	1.35E+05	9.35E+03
B	3		6: 43 58 42 39 38 36	99	1.96E+06	1.35E+05
B	4		10: 43 58 59 42 41 39 38 37 36 45	99	2.96E+06	2.05E+05
C	1			87	4.25E+04	2.94E+03
C	2		2: 43 42	90	6.65E+04	4.60E+03
C	3		3: 43 58 42	88	7.06E+04	4.88E+03
2-methyl-2-propanamine	75-64-9	1.67					A	2		1: 58	89	7.42E+04	
A	6		3: 60 53 36	79	9.02E+04	
B	1		3: 42 41 55	76	9.02E+04	
C	3		2: 58 42	70	3.20E+04	
+Acetic anhydride	108-24-7	1.70		Sharp, Vinegar		5.89E-01	A	1		1: 43	66	9.90E+03	1.68E+04
A	2		1: 43	72	2.31E+05	3.93E+05
A	3			66	1.47E+04	2.50E+04
A	4		2: 43 41	81	3.73E+04	6.33E+04
A	5		5: 43 42 39 41 37	80	4.76E+05	8.09E+05
A	6			65	2.59E+03	4.40E+03
A	7		2: 43 85	77	5.45E+04	9.26E+04
B	4		2: 42 43	79	8.59E+04	1.46E+05
C	3		2: 43 41	70	3.38E+04	5.74E+04
Isobutyraldehyde	78-84-2	1.76	Pungent, Malt, Green	Spicy		4.07E-02	A	1			78	2.20E+07	5.40E+08
A	2		13: 43 41 57 72 39 55 56 38 40 73 62 66 65	78	2.02E+07	4.96E+08
A	3		3: 53 73 61	80	1.63E+06	3.99E+07
A	6			73	6.15E+03	1.51E+05
A	7			88	1.52E+04	3.73E+05
B	1			93	3.08E+04	7.55E+05
B	2		7: 42 41 72 53 55 56 38	77	7.00E+05	1.72E+07
B	3			85	7.70E+03	1.89E+05
B	4		4: 42 43 57 72	75	6.45E+04	1.58E+06
+Methyl acetate	79-20-9	1.77		Ethereal			A	1			81	2.05E+04	
A	4		1: 43	97	2.81E+05	
A	6		7: 43 74 59 42 45 72 44	94	1.54E+06	
B	3		5: 43 41 59 73 75	99	4.56E+05	
B	4		4: 43 74 42 59	95	1.24E+05	
Cyclohexene	110-83-8	2.02				3.63E-01	A	7		2: 67 82	70	2.95E+04	8.12E+04
Methacrolein	78-85-3	2.14		Wild hyacinth foliage			A	7			87	3.42E+04	
+Butyraldehyde	123-72-8	2.16	Pungent, Green	Pungent, Cocoa, Musty, Green, Malty, Bread			A	6			72	4.12E+04	
B	1		3: 41 44 72	73	1.92E+04	
B	3			89	2.08E+04	
B	4			91	2.41E+04	
+2-butenal	4170-30-3	2.31		Flower		1.35E-01	A	7			79	3.42E+04	2.54E+05
A	4		1: 43	68	3.13E+05	4.03E+04
							A	6			79	1.13E+04	1.46E+03
							A	7		1: 43	79	2.25E+04	2.90E+03
B	1		1: 43	76	1.16E+05	1.49E+04
B	3		3: 72 43 127	75	3.07E+04	3.96E+03
C	1		2: 43 72	79	1.06E+04	1.37E+03
methylhydrazine	60-34-4	2.32					A	3			79	2.05E+04	
Diazomethane	334-88-3	2.33					A	7		3: 41 42 59	71	3.00E+04	
B	3		2: 40 42	66	1.01E+05	
+Isopropyl alcohol	67-63-0	2.33		Alcohol, Musty, Woody		1.02E+01	A	1		3: 45 43 41	77	1.50E+05	1.46E+04
A	3		2: 45 42	68	5.38E+04	5.26E+03
A	6		3: 45 44 72	75	1.43E+05	1.40E+04
B	3			68	7.49E+04	7.32E+03
C	1		4: 44 90 38 37	70	6.13E+05	5.99E+04
C	2		15: 45 57 44 47 46 42 72 56 73 39 60 89 71 38 74	65	8.30E+06	8.12E+05
C	3		1: 45	65	1.24E+04	1.21E+03
+Formic acid	64-18-6	2.33		Acetic		2.82E+01	A	1		3: 46 42 45	69	1.38E+05	4.91E+03
A	2		1: 46	79	6.39E+04	2.27E+03
A	3			79	4.06E+04	1.44E+03
A	7		6: 45 46 39 42 41 47	67	4.35E+05	1.55E+04
B	1		4: 46 45 39 42	67	1.45E+05	5.14E+03
B	2		2: 45 46	77	4.31E+04	1.53E+03
Nitrogen dioxide	10102-44-0	2.34				1.86E-01	A	1		1: 46	76	2.63E+04	1.41E+05
A	2		1: 46	76	4.35E+04	2.34E+05
+Ethanol	64-17-5	2.34	Sweet	Alcoholic		2.88E+01	A	1			95	1.29E+05	4.47E+03
A	2			94	1.20E+05	4.15E+03
A	3			95	7.50E+04	2.60E+03
A	6			72	1.69E+05	5.86E+03
A	7		6: 45 46 39 42 41 47	99	3.01E+05	1.04E+04
B	1		4: 46 45 39 42	84	9.90E+04	3.43E+03
B	2		2: 45 46	78	4.31E+04	1.49E+03
B	3		4: 43 207 42 46	79	5.88E+04	2.04E+03
B	4			92	5.54E+04	1.92E+03
+Methylene chloride	75-09-2	2.42				2.82E+01	A	1		2: 86 39	94	1.76E+05	6.23E+03
A	2		3: 51 48 47	97	2.98E+05	1.06E+04
A	4		5: 84 51 88 42 50	97	3.88E+05	1.38E+04
A	5		2: 47 49	98	2.81E+05	9.98E+03
A	6			92	2.13E+04	7.56E+02
A	7		5: 84 39 86 88 47	95	7.08E+04	2.51E+03
B	3			91	1.67E+04	5.92E+02
B	4		7: 84 49 88 51 47 83 48	97	1.62E+05	5.74E+03
Amitrole	61-82-5	2.44					B	4		1: 84	67	4.85E+04	
Allyl alcohol	107-18-6	2.75		Pungent, Mustard		2.69E-01	A	6			71	1.94E+04	7.19E+04
+Methylbutanal	590-86-3	2.75	Malt	Ethereal, Aldehydic, Chocolate, Peach, Fatty	1.00E+00	2.24E-03	B	3			68	2.92E+04	1.30E+07
Allyl alcohol	107-18-6	2.75		Pungent, Mustard		2.69E-01	B	3			75	2.92E+04	1.08E+05
Acetonitrile	75-05-8	3.28			9.77E+01		B	1			96	1.18E+05	1.20E+03
B	2			96	9.20E+04	9.42E+02
Chloroform	67-66-3	3.78					A	1		2: 85 83	78	3.97E+04	
A	2			76	1.45E+04	
A	4			79	1.70E+04	
A	5			84	2.62E+04	
A	6			86	2.07E+04	
B	4		2: 83 47	79	7.69E+04	
Propyl formate	110-74-7	3.91		Sweet, Ethereal, Green, Rum, Fruity, Berry		3.39E+00	A	7		1: 42	72	1.56E+05	4.59E+04
Hydrazine	302-01-2	3.92				3.00E+00	A	1		1: 33	77	2.35E+03	7.85E+02
A	2		1: 33	77	1.56E+03	5.21E+02
A	3		1: 33	77	9.74E+02	3.25E+02
A	7		3: 33 45 37	77	4.44E+03	1.48E+03
B	1		1: 33	77	3.54E+03	1.18E+03
B	4		1: 33	76	8.26E+02	2.75E+02
3-pentanol	584-02-1	3.92	Fruit	Herbal		4.68E-01	A	1			69	1.14E+05	2.43E+05
A	2		3: 60 59 45	68	1.06E+05	2.27E+05
A	3			67	6.67E+04	1.43E+05
A	7			71	1.21E+05	2.58E+05
B	1			70	1.01E+05	2.17E+05
B	3		3: 59 60 53	77	7.79E+05	1.67E+06
B	4		9: 59 42 60 41 57 39 58 40 36	75	2.14E+06	4.58E+06
+1,1-dimethyl-hydrazine	57-14-7	3.92					A	1		1: 42	76	6.10E+04	
A	2		3: 60 59 45	80	1.06E+05	
A	3		1: 42	76	1.59E+05	
A	7			83	1.41E+05	
B	1		2: 59 45	77	6.29E+04	
B	3		3: 59 60 33	80	1.87E+05	
B	4		9: 59 42 60 41 57 39 58 40 36	80	6.00E+05	
Ethylenediamine	107-15-3	3.92					A	1			79	7.94E+04	
A	2			75	8.76E+04	
A	3		3: 42 60 59	71	2.81E+04	
A	7			75	1.41E+05	
B	1			75	1.01E+05	
B	3		3: 59 60 53	71	4.76E+05	
B	4		7: 41 38 60 61 33 44 58	79	5.13E+05	
tert-butanol	75-65-0	3.93		Camphor			A	2			70	1.53E+04	
B	3		3: 59 60 53	77	7.79E+05	
B	4		9: 59 42 60 41 57 39 58 40 36	74	2.14E+06	
Methyl formate	107-31-3	3.93		Fruity, Plum		9.33E+01	A	1		1: 33	79	5.81E+03	6.22E+01
A	4		1: 60	72	3.42E+03	3.67E+01
B	4		7: 41 38 60 61 33 44 58	71	5.62E+05	6.02E+03
Propylamine	107-10-8	3.94		Ammoniacal		1.10E-02	A	2			76	5.74E+04	5.23E+06
B	4		9: 59 42 60 41 57 39 58 40 36	73	2.12E+06	1.94E+08
Tetrahydrofurfuryl acetate	637-64-9	4.07		Sweet, Fruity, Brown, Rum, Ether, Caramel			A	6			70	2.00E+04	
B	3		4: 71 55 43 67	67	1.57E+04	
+Phenylethyl alcohol	60-12-8	5.06	Honey, Spice, Rose, Lilac	Floral		1.70E-02	A	2			74	9.14E+04	5.38E+06
+Toluene	108-88-3	5.07	Paint	Sweet		1.55E+00	A	1			81	1.39E+04	8.98E+03
A	2			96	9.14E+04	5.90E+04
B	1		1: 91	79	5.82E+04	3.76E+04
+Pentanal	110-62-3	5.97	Almond, Malt, Pungent	Fermented		6.03E-03	B	3		3: 44 41 58	70	3.47E+04	5.76E+06
+Hexanal	66-25-1	5.99	Grass, Tallow, Fat	Green	4.00E-03	1.38E-02	A	6			85	4.12E+04	2.98E+06
A	7			84	3.44E+04	2.49E+06
B	1		7: 43 41 72 55 45 207 82	90	1.22E+05	8.87E+06
B	2		11: 82 44 55 41 72 39 45 43 81 58 53	94	5.45E+05	3.95E+07
B	3			73	2.30E+04	1.66E+06
Glutaraldehyde	111-30-8	6.02					B	1			69	1.15E+05	
B	2		11: 82 44 55 41 72 39 45 43 81 58 53	70	5.45E+05	
+1-butanol	71-36-3	6.09	Medicine, Fruit	Fermented		4.90E-01	A	3		2: 56 41	79	1.25E+05	2.54E+05
A	4			77	1.82E+04	3.72E+04
A	6		4: 43 39 56 42	79	3.76E+04	7.67E+04
A	7		3: 41 39 56	83	3.18E+04	6.48E+04
B	1		3: 56 55 39	92	1.21E+05	2.47E+05
B	2		2: 57 208	66	2.51E+05	5.12E+05
B	3		5: 56 43 57 39 72	97	9.87E+05	2.01E+06
B	4		12: 41 43 55 42 45 39 38 40 33 37 73 49	97	1.44E+06	2.95E+06
Butyl formate	592-84-7	6.09		Fruity			A	3		2: 56 41	67	9.56E+04	
A	7		3: 41 39 56	65	2.32E+04	
B	3		7: 39 43 56 40 57 41 44	82	9.78E+05	
B	4		16: 56 41 43 55 39 40 46 57 73 38 45 74 51 49 54 50	85	3.94E+06	
+Isobutanol	78-83-1	6.10	Wine, Solvent, Bitter	Ethereal, Winey			B	4		2: 59 37	85	2.76E+05	
Propanoic acid, anhydride	123-62-6	6.46					A	1		1: 57	67	1.65E+03	
A	2		1: 57	67	3.80E+03	
A	3			68	4.04E+03	
A	4		3: 57 85 34	68	7.52E+03	
A	6		1: 57	76	3.42E+04	
A	7		1: 57	68	1.34E+04	
B	2			69	4.60E+03	
B	3			77	1.89E+04	
C	1		1: 57	66	3.20E+03	
C	2		1: 57	66	4.68E+03	
C	3		1: 57	66	8.12E+03	
4-methyl-3-penten-2-one	141-79-7	6.66	Sweet, Chemical	Pungent, Earthy, Vegetable, Acrylic		5.62E-02	B	1		2: 98 83	73	3.78E+04	6.72E+05
B	3			94	1.03E+05	1.83E+06
B	4		4: 55 42 63 77	97	6.77E+05	1.20E+07
2,2׳-Bioxirane	1464-53-5	6.66					B	3		3: 55 51 43	65	9.31E+04	
α-angelica lactone	591-12-8	6.66					B	3		3: 55 51 43	82	1.17E+05	
+Isoamyl alcohol	123-51-3	7.52	Whiskey, Malt, Burnt	Fusel oil, Alcoholic, Whiskey, Fruity, Banana		4.47E-02	A	6			73	3.77E+04	8.43E+05
B	3			75	7.91E+04	1.77E+06
Amyl alcohol	71-41-0	7.52	Balsamic	Fusel, Oil, Sweet, Balsam		4.68E-01	A	6			78	3.77E+04	8.05E+04
B	3			79	7.91E+04	1.69E+05
2-isopropenyl-3-methylpyrazine	145984-65-2	7.67					A	7		4: 135 75 134 133	73	1.03E+05	
α-phellandrene	99-83-2	7.89	Turpentine, Mint, Spice	Terpenic			A	1		20: 77 40 80 43 121 94 78 92 38 107 136 42 82 90 50 33 137 115 135 117	81	1.00E+07	
A	2		5: 91 107 93 136 92	95	7.06E+05	
A	3		6: 105 107 93 77 81 54	74	3.57E+05	
A	5		13: 94 91 93 55 51 136 92 79 121 77 108 103 122	90	8.87E+04	
A	6		4: 93 55 105 78	90	9.41E+04	
A	7		11: 92 136 91 93 108 78 39 77 107 106 66	79	1.55E+06	
B	2		20: 93 39 67 136 94 77 79 78 92 80 53 41 81 68 137 55 63 95 52 69	74	1.37E+06	
B	3		2: 91 93	82	1.77E+04	
B	4			85	3.25E+04	
C	1			90	8.81E+04	
C	2			86	3.75E+04	
C	3		7: 136 93 91 92 103 77 94	89	8.42E+04	
α-pinene	80-56-8	7.90	Pine, Turpentine	Herbal		6.92E-01	A	1		12: 79 93 106 91 78 41 136 51 94 92 77 67	93	1.05E+06	1.52E+06
A	2			97	6.09E+06	8.80E+06
A	3		10: 93 91 121 77 43 81 106 94 39 53	92	2.14E+05	3.09E+05
A	5		8: 93 81 68 107 43 105 95 78	93	3.65E+05	5.28E+05
A	6			93	1.61E+05	2.33E+05
A	7		11: 92 136 91 93 108 78 39 77 107 106 66	97	4.88E+05	7.05E+05
B	1		11: 92 81 78 39 41 65 107 281 80 122 69	98	1.79E+06	2.58E+06
B	2		20: 93 39 67 136 94 77 79 78 92 80 53 41 81 68 137 55 63 95 52 69	98	1.24E+06	1.79E+06
B	3			88	9.49E+04	1.37E+05
B	4			83	3.25E+04	4.69E+04
C	1			71	5.23E+04	7.56E+04
C	2			75	3.75E+04	5.42E+04
C	3		7: 136 93 91 92 103 77 94	70	8.42E+04	1.22E+05
Betahistine	5638-76-6	7.90					A	1		3: 65 74 104	65	2.02E+07	
A	3			69	4.37E+04	
A	4			67	1.03E+04	
A	5		8: 136 93 80 43 41 106 65 94	70	5.56E+05	
A	6		6: 136 94 79 106 93 121	73	4.42E+05	
A	7			68	5.61E+05	
B	1		18: 77 79 68 80 53 52 121 136 106 105 43 41 64 51 103 66 81 54	67	1.29E+06	
Conessine	546-06-5	8.31					B	3		2: 71 84	69	5.94E+03	
B	4		1: 84	74	5.31E+04	
2-formyl pyrrole	1003-29-8	9.09		Musty, Beefy, Coffee			C	3		3: 95 94 81	67	7.92E+03	
1,4-dimethoxybenzene	150-78-7	9.19		Sweet, Green, New mown hay, Fennel			C	1			67	1.39E+04	
C	2			66	2.81E+04	
+α-ionol	25312-34-9	9.20		Ionone, Tropical, Sweet, Floral, Violet, Woody			C	1			73	2.42E+04	
C	3		7: 138 95 82 80 55 45 140	68	1.92E+05	
Menthyl acetate	16409-45-3	9.20		Tea cooling, Minty, Fruity, Berry		6.17E+00	C	1		5: 138 94 123 95 79	74	1.46E+05	2.37E+04
C	2		2: 138 96	79	5.85E+04	9.49E+03
C	3			77	2.29E+04	3.71E+03
4-methyl guaiacol	93-51-6	9.20		Spicy			C	1		5: 138 94 123 95 79	74	1.18E+05	
2-acetyl-6-methyl pyrazine	22047-26-3	9.26		Roasted coffee, Cocoa, Popcorn			B	3		3: 93 136 41	70	1.67E+04	
C	2		15: 93 136 39 80 94 78 67 108 102 104 120 38 75 54 49	65	3.00E+07	
Tricyclene	508-32-7	9.30					A	2		6: 136 133 92 78 107 40	81	3.13E+05	
A	6		6: 136 94 79 106 93 121	71	4.42E+05	
2-indanone	615-13-4	9.47					B	1		6: 104 77 103 39 51 102	66	1.82E+05	
+Styrene	100-42-5	9.48	Balsamic, Gasoline	Balsamic		1.45E-01	B	1		6: 104 77 103 39 51 102	97	1.82E+05	1.26E+06
B	2			81	2.19E+04	1.52E+05
β-pinene	18172-67-3	9.90	Pine, Resin, Turpentine	Terpenic			A	1		11: 136 52 128 81 119 78 90 56 83 59 55	96	2.64E+07	
A	2		18: 69 41 79 53 39 94 67 80 70 107 66 51 117 137 52 104 37 59	97	1.09E+07	
A	3			93	7.03E+05	
A	4			67	2.39E+04	
A	5		5: 69 93 121 51 94	80	5.33E+05	
A	6			73	8.95E+05	
A	7			92	5.61E+05	
B	1		20: 93 69 41 91 39 68 51 92 79 136 77 67 65 94 53 54 107 82 137 52	96	3.92E+06	
B	2		20: 69 53 77 78 39 94 121 70 55 42 68 65 89 67 52 40 51 105 66 56	95	5.72E+06	
B	3			75	3.51E+04	
+Myrcene	123-35-3	9.94	Balsamic, Must, Spice	Peppery, Terpene, Spicy, Balsam, Plastic	1.30E-02		A	1		12: 41 92 43 120 80 40 53 51 55 79 52 78	88	1.86E+06	1.43E+08
A	2		1: 38	92	1.36E+06	1.05E+08
A	3		12: 93 81 41 94 77 43 91 121 70 79 51 106	94	1.37E+06	1.05E+08
A	5			92	2.88E+05	2.22E+07
A	7			94	6.11E+05	4.70E+07
B	1		20: 93 69 41 91 39 68 51 92 79 136 77 67 65 94 53 54 107 82 137 52	97	3.92E+06	3.02E+08
B	2		20: 69 53 77 78 39 94 121 70 55 42 68 65 89 67 52 40 51 105 66 56	96	5.72E+06	4.40E+08
B	3		3: 93 92 41	71	3.05E+04	2.34E+06
DL-menthol	89-78-1	10.34		Peppermint, Cool, Woody	4.17E-02		C	1			67	1.01E+05	2.42E+06
C	3		7: 138 95 82 80 55 45 140	66	6.55E+04	1.57E+06
+(±)-menthol	1490-04-6	10.34		Minty		4.17E-02	C	1			70	1.01E+05	2.42E+06
C	2		9: 95 138 139 96 94 67 109 123 68	70	1.39E+05	3.34E+06
C	3			69	1.90E+05	4.56E+06
+o-dimethyl hydroquinone	91-16-7	10.34		Vanilla			C	3		9: 95 94 138 96 123 67 53 81 79	69	3.63E+05	
(+)-carvomenthene	1195-31-9	10.34					C	1			85	1.01E+05	
C	2			85	8.21E+04	
C	3			86	1.90E+05	
Menthol	15356-70-4	10.36			4.17E-02		C	3			66	8.02E+04	1.92E+06
2,4,6-trimethylphenol	527-60-6	10.48		Phenolic			C	1		6: 57 39 107 135 116 52	82	4.11E+05	
+α-terpinene	99-86-5	10.50	Lemon	Woody			A	1		3: 121 75 68	66	1.37E+06	
A	2		20: 136 121 93 91 79 77 105 39 51 41 64 120 53 107 106 55 95 50 40 116	96	4.75E+06	
A	7		13: 121 78 136 68 103 117 80 52 51 77 106 107 81	80	2.20E+05	
B	1		3: 136 91 107	78	4.82E+04	
C	1		20: 136 93 53 91 78 41 107 122 137 77 79 105 92 119 50 39 65 108 115 90	97	2.04E+06	
C	2			97	1.40E+06	
C	3		20: 91 93 79 107 136 92 106 77 95 65 89 51 43 108 137 94 102 68 115 50	98	1.71E+06	
(+)-4-Carene	29050-33-7	10.50			4.00E+00		A	1		7: 93 119 121 137 53 105 136	94	3.68E+05	9.21E+04
A	2		20: 136 121 93 91 79 77 105 39 51 41 64 120 53 107 106 55 95 50 40 116	99	4.75E+06	1.19E+06
A	3			91	3.19E+04	7.97E+03
A	5		5: 105 119 121 80 136	67	7.28E+04	1.82E+04
A	7		13: 121 78 136 68 103 117 80 52 51 77 106 107 81	82	2.20E+05	5.49E+04
B	1			84	2.29E+04	5.73E+03
C	1		20: 136 93 53 91 78 41 107 122 137 77 79 105 92 119 50 39 65 108 115 90	98	2.04E+06	5.10E+05
C	2			98	1.40E+06	3.50E+05
C	3		20: 121 136 122 103 78 77 105 80 41 106 107 39 43 94 120 115 52 135 67 54	98	1.10E+06	2.74E+05
Furfurylmethylamphetamine	13445-60-8	10.50					A	1		1: 81	73	5.34E+04	
+Phenylacetic acid	103-82-2	10.53	Honey, Flower	Sweet, Honey, Floral, Honeysuckle, Sour, Waxy, Civet			A	2		3: 91 136 43	70	7.97E+04	
+1-hexanol	111-27-3	10.73	Resin, Flower, Breen	Herbal		4.37E-02	A	6			87	5.03E+04	1.15E+06
Diacetone alcohol	123-42-2	10.78				8.91E-01	A	7		2: 59 43	77	5.51E+04	6.18E+04
B	3			87	2.54E+05	2.85E+05
B	4		9: 43 59 101 39 83 55 61 40 45	92	1.75E+06	1.96E+06
(1R)-(+)-trans-isolimonene	5113-87-1	10.85					B	4		8: 79 121 136 105 94 95 108 81	71	6.65E+04	
2,2,5-trimethylhexane	3522-94-9	10.88					A	4		4: 56 57 71 136	80	1.28E+05	
+Limonene	138-86-3	10.89	Lemon, Orange	Citrus	1.00E-02	4.37E-01	A	1		20: 92 105 80 51 117 66 137 122 62 81 64 54 104 63 76 108 103 134 38 43	95	3.33E+07	7.64E+07
A	2		20: 68 93 67 94 136 59 107 91 53 39 81 95 105 55 65 66 119 137 52 96	95	2.21E+07	5.05E+07
A	3		20: 68 79 93 67 107 115 94 92 77 63 136 39 80 41 91 108 69 95 54 137	95	1.97E+06	4.51E+06
A	4		20: 68 92 93 41 67 39 57 71 65 77 55 79 85 94 53 136 91 56 121 191	82	4.35E+05	9.97E+05
A	6		8: 92 67 93 65 136 80 107 39	87	2.06E+05	4.71E+05
A	7		6: 68 92 80 136 69 41	90	1.71E+05	3.92E+05
B	1			95	2.16E+06	4.94E+06
B	2			95	1.71E+06	3.93E+06
B	3			90	1.76E+05	4.03E+05
B	4		3: 67 121 77	76	8.21E+04	1.88E+05
C	1		16: 93 67 69 107 39 121 92 136 52 122 41 137 42 77 55 51	93	3.17E+05	7.26E+05
C	2		7: 68 91 136 67 121 65 69	87	1.62E+05	3.71E+05
C	3			80	3.86E+04	8.84E+04
+Camphene	79-92-5	10.93	Camphor	Woody			A	1		20: 92 105 80 51 117 66 137 122 62 81 64 54 104 63 76 108 103 134 38 43	89	3.33E+07	
A	2		20: 68 93 67 94 136 59 107 91 53 39 81 95 105 55 65 66 119 137 52 96	89	2.21E+07	
A	3		20: 68 79 93 67 107 115 94 92 77 63 136 39 80 41 91 108 69 95 54 137	89	1.97E+06	
A	4		20: 68 92 93 41 67 39 57 71 65 77 55 79 85 94 53 136 91 56 121 191	78	4.35E+05	
A	5		12: 91 53 67 65 121 107 80 105 93 77 41 95	87	4.38E+05	
A	6		8: 92 67 93 65 136 80 107 39	82	2.06E+05	
A	7		7: 79 68 136 107 92 95 91	84	1.33E+05	
B	1			90	2.16E+06	
B	2			90	1.71E+06	
B	3			85	1.76E+05	
B	4		8: 79 121 136 105 94 95 108 81	79	3.71E+04	
C	1			88	2.97E+05	
C	2		11: 93 94 120 51 122 77 65 104 108 52 103	65	2.52E+05	
C	3			82	8.67E+07	
Eucalyptol	470-82-6	10.97	Mint, Sweet	Eucalyptus, Herbal, Camphor		1.62E-02	A	6			80	1.58E+05	9.75E+06
B	3			70	1.03E+05	6.36E+06
N-Benzyl-2-phenethylamine	3647-71-0	11.32					B	3		2: 120 91	75	1.22E+04	
Phenyl propane	103-65-1	11.32					B	3		2: 120 91	70	1.22E+04	
3-ethyl-o-xylene	933-98-2	11.35					A	2		13: 91 119 78 77 105 55 103 50 104 120 135 133 63	81	8.85E+05	
m-cymene	535-77-3	11.36					A	1			96	3.98E+05	
A	2			98	1.76E+06	
A	3		2: 119 91	73	3.59E+04	
A	4		2: 119 65	71	4.21E+04	
A	6			92	4.31E+04	
A	7			86	3.49E+04	
B	1			91	5.27E+04	
B	3		3: 119 91 134	93	9.48E+04	
C	1		19: 64 90 106 76 59 49 133 128 66 85 107 129 101 126 113 67 73 111 130	99	6.79E+07	
C	2			99	3.74E+07	
C	3		20: 119 134 91 77 135 93 92 51 78 116 58 50 128 52 86 87 129 101 131 126	97	3.94E+07	
+p-cymene	99-87-6	11.36	Solvent, Gasoline, Citrus	Terpenic		2.14E-03	A	1			93	3.98E+05	1.86E+08
A	2			95	1.76E+06	8.22E+08
A	3		1: 119	74	5.38E+04	2.52E+07
A	4		2: 119 65	70	5.56E+04	2.60E+07
A	6			91	4.31E+04	2.01E+07
A	7			85	3.49E+04	1.63E+07
B	1			89	5.27E+04	2.46E+07
B	3		3: 119 91 134	91	9.48E+04	4.44E+07
C	1		19: 64 90 106 76 59 49 133 128 66 85 107 129 101 126 113 67 73 111 130	97	6.79E+07	3.18E+10
C	2			97	3.74E+07	1.75E+10
C	3		20: 119 134 91 77 135 93 92 51 78 116 58 50 128 52 86 87 129 101 131 126	93	3.94E+07	1.84E+10
1,2,3,4-tetramethylbenzene	488-23-3	11.36		Gasoline, Sweet		2.63E-02	A	1			91	3.98E+05	1.51E+07
A	2		14: 119 134 117 118 39 135 103 89 116 133 41 78 64 51	94	1.33E+06	5.06E+07
A	3		2: 134 119	72	4.73E+04	1.80E+06
A	4		3: 119 117 63	67	2.58E+04	9.82E+05
A	5		2: 119 134	67	8.39E+03	3.19E+05
A	6		5: 119 134 120 117 57	86	1.25E+05	4.77E+06
A	7			80	3.49E+04	1.33E+06
B	1			85	5.27E+04	2.00E+06
B	3		3: 119 91 134	87	9.48E+04	3.60E+06
C	1		19: 64 90 106 76 59 49 133 128 66 85 107 129 101 126 113 67 73 111 130	95	6.79E+07	2.58E+09
C	2			95	3.74E+07	1.42E+09
C	3		20: 119 134 91 77 135 93 92 51 78 116 58 50 128 52 86 87 129 101 131 126	93	3.94E+07	1.50E+09
1-ethyl-2,4-dimethylbenzene	874-41-9	11.36					A	1		5: 93 78 119 104 106	76	1.24E+05	
A	3		1: 119	78	5.38E+04	
A	4		2: 119 65	72	5.56E+04	
A	6		5: 119 134 120 117 57	84	1.23E+05	
B	1		3: 134 120 77	79	7.74E+04	
B	3		2: 119 120	76	6.36E+04	
1-phenyl-1-decanone	6048-82-4	11.40					A	2		6: 57 63 117 58 120 105	71	2.12E+05	
N,N-dimethylbenzenamine	121-69-7	11.40					A	2		6: 57 63 117 58 120 105	71	2.12E+05	
Isodurene	527-53-7	11.40					A	1		3: 57 119 134	79	2.50E+04	
A	2		4: 119 39 134 193	75	3.12E+04	
A	4		3: 119 117 63	69	2.60E+04	
B	1		2: 119 134	67	7.78E+04	
C	1		20: 119 134 91 105 103 104 39 75 128 50 53 52 90 76 38 94 106 114 85 98	77	5.94E+07	
1-(3-methylphenyl)-ethanone	585-74-0	11.41					A	1		5: 93 78 119 104 106	83	7.20E+04	
A	3		2: 119 91	71	3.59E+04	
B	1		3: 91 120 134	76	6.51E+04	
Dihydromethylcyclopentapyrazine	23747-48-0	11.41	Roast, Nut	Earthy, Baked potato, Peanut, Roasted			A	1		3: 57 119 134	75	1.56E+05	
A	2		4: 119 39 134 193	69	3.12E+04	
C	1		19: 64 90 106 76 59 49 133 128 66 85 107 129 101 126 113 67 73 111 130	70	2.87E+07	
1-ethyl-3,5-dimethylbenzene	934-74-7	11.46					A	2		8: 134 119 116 117 62 102 57 71	68	3.87E+05	
C	1		20: 119 134 91 105 103 104 39 75 128 50 53 52 90 76 38 94 106 114 85 98	82	6.16E+07	
+Methylisohexenyl ketone	110-93-0	11.51	Pepper, Mushroom, Rubber	Citrus		3.80E-02	A	4			93	3.18E+05	8.35E+06
A	6		9: 52 68 65 57 45 77 54 84 50	98	4.30E+06	1.13E+08
A	7			80	4.51E+04	1.19E+06
B	3		4: 65 70 82 97	98	3.30E+06	8.67E+07
B	4		7: 93 108 67 117 55 68 126	94	3.97E+05	1.04E+07
4-ethyl-1,2-dimethylbenzene	934-80-5	11.57					A	2		8: 134 119 116 117 62 102 57 71	67	3.91E+05	
+δ-3-carene	13466-78-9	11.57	Lemon, Resin	Citrus	4.00E+00		A	1		4: 108 91 43 105	78	7.76E+04	1.94E+04
A	2		8: 134 119 116 117 62 102 57 71	97	3.80E+06	9.51E+05
A	3			90	7.79E+04	1.95E+04
A	5		9: 107 93 92 136 80 118 65 120 79	71	3.01E+05	7.53E+04
A	6			73	9.13E+05	2.28E+05
A	7		19: 92 79 94 105 95 91 148 63 65 204 120 41 123 82 135 78 66 39 128	70	1.42E+06	3.56E+05
B	1		11: 137 79 93 136 92 107 94 77 78 81 53	96	3.24E+05	8.11E+04
B	2		7: 79 43 67 51 136 40 105	75	3.15E+05	7.88E+04
B	3			72	8.48E+05	2.12E+05
B	4			71	2.10E+05	5.25E+04
C	1		4: 90 41 122 107	85	9.59E+06	2.40E+06
C	2		10: 106 122 108 138 135 94 64 68 82 63	90	2.21E+06	5.52E+05
C	3		20: 91 93 79 107 136 92 106 77 95 65 89 51 43 108 137 94 102 68 115 50	72	1.82E+06	4.54E+05
Sabinene	3387-41-5	11.59	Pepper, Turpentine, Wood	Woody			A	1		20: 77 40 80 43 121 94 78 92 38 107 136 42 82 90 50 33 137 115 135 117	79	9.81E+06	
A	2		11: 136 105 92 67 79 43 68 94 51 106 138	88	1.53E+06	
A	3			90	7.79E+04	
A	7		7: 91 67 107 108 41 94 63	69	1.09E+05	
B	1		18: 77 79 68 80 53 52 121 136 106 105 43 41 64 51 103 66 54 81	75	1.30E+06	
γ-terpinene	99-85-4	11.79	Gasoline, Turpentine	Terpenic			A	2			85	5.28E+04	
A	7		7: 77 107 80 121 92 137 63	69	1.64E+05	
C	1		14: 91 136 105 79 78 53 76 80 137 55 81 75 68 127	98	1.32E+08	
C	2		18: 91 79 43 107 119 51 103 117 66 55 88 74 135 129 42 123 101 87	99	1.25E+08	
C	3		16: 93 91 121 105 41 43 63 122 52 81 76 102 38 42 127 120	99	8.67E+07	
+Terpinolene	586-62-9	11.83	Pine, Plastic	Herbal	2.00E-01		A	1		11: 136 52 128 81 119 78 90 56 83 59 55	73	3.87E+06	1.94E+07
A	2		20: 136 121 93 91 79 77 105 39 51 41 64 120 53 107 106 55 95 50 40 116	90	6.01E+06	3.00E+07
A	3			87	3.19E+04	1.59E+05
C	1		3: 137 67 104	82	4.17E+05	2.09E+06
C	2		18: 91 79 43 107 119 51 103 117 66 55 88 74 135 129 42 123 101 87	95	1.25E+08	6.26E+08
C	3		16: 93 91 121 105 41 43 63 122 52 81 76 102 38 42 127 120	95	8.67E+07	4.33E+08
+Ethyl benzene	100-41-4	11.84				2.88E+00	C	1		14: 91 136 105 79 78 53 76 80 137 55 81 75 68 127	67	9.64E+07	3.34E+07
C	2		18: 91 79 43 107 119 51 103 117 66 55 88 74 135 129 42 123 101 87	71	7.80E+07	2.70E+07
+Acetic acid	64-19-7	12.23	Sour	Acidic		1.45E-01	A	6		8: 43 60 45 42 41 44 40 59	100	2.76E+07	1.91E+08
A	7		3: 43 44 207	100	7.66E+05	5.30E+06
B	1		3: 45 43 60	90	1.91E+04	1.32E+05
B	3		4: 43 45 60 42	98	1.62E+07	1.12E+08
N-methyl-N-nitroso urea	684-93-5	12.26					A	7		4: 43 60 42 44	70	1.71E+05	
(z)-rose oxide	16409-43-1	12.28		Green, Red rose, Spic, Fresh geranium			C	2		14: 69 139 96 97 83 70 95 55 140 207 154 67 71 66	66	2.19E+05	
C	3		4: 139 140 96 84	68	2.25E+05	
Phenetole	103-73-1	12.52					A	2		7: 122 107 68 51 50 94 117	70	1.18E+05	
2-hydroxyacetophenone	118-93-4	12.53		Phenolic			A	1			86	7.20E+04	
A	2		5: 136 107 137 93 122	85	7.49E+04	
A	3			80	1.89E+04	
A	4		2: 121 136	75	5.91E+04	
A	6		2: 121 136	74	9.22E+03	
A	7		7: 43 136 121 81 92 53 91	67	2.20E+04	
C	1		13: 121 93 41 122 136 67 55 108 106 92 104 53 94	78	8.16E+05	
1-methyl-2-propyl benzene	1074-17-5	12.63					C	1		2: 134 105	73	1.91E+04	
2-phenyl propionaldehyde	93-53-8	12.63		Fresh, Sharp, Green, Hyacinth, Leaf, Lilac			C	1		2: 134 105	66	1.91E+04	
+o-xylene	95-47-6	13.07	Geranium	Geranium		8.51E-01	A	2		5: 77 134 106 119 52	73	9.60E+04	1.13E+05
+p-xylene	106-42-3	13.08				4.90E-01	A	2			81	4.01E+04	8.19E+04
4-methylphenethylamine	3261-62-9	13.08					A	2			69	4.01E+04	
2,3-dimethyl-cyclohexanol	1502-24-5	13.19					B	3			68	1.94E+04	
Fenchone	1195-79-5	13.47				9.33E-02	A	6		6: 81 69 152 53 80 67	95	2.96E+05	3.17E+06
B	3		4: 41 39 109 77	91	1.41E+05	1.51E+06
C	1		19: 81 41 53 55 79 39 82 91 80 137 67 70 42 105 123 85 38 153 77	98	1.36E+06	1.46E+07
C	2		13: 153 152 80 55 77 78 91 42 71 66 52 40 123	99	1.43E+06	1.53E+07
C	3		20: 81 69 152 67 80 41 66 68 82 39 109 72 91 52 55 137 97 42 153 40	98	1.50E+06	1.60E+07
Linalool oxide	5989-33-3	13.67	Flower, Wood	Earthy, Floral, Sweet, Woody			A	5		11: 207 266 83 70 79 55 112 67 85 53 97	65	1.91E+05	
A	6			83	8.06E+04	
A	7		19: 93 55 111 70 92 71 94 43 67 81 83 68 91 69 84 74 57 137 82	80	3.23E+05	
B	3		5: 111 81 71 95 93	82	8.43E+04	
1,3-diethylbenzene	141-93-5	13.81					A	2		14: 105 93 94 137 81 53 65 119 77 120 82 135 51 39	68	3.70E+05	
+2-ethylhexanol	104-76-7	13.81	Rose, Green	Citrus		2.45E-01	A	5			87	1.48E+05	6.05E+05
A	6			85	9.88E+04	4.03E+05
A	7		7: 84 41 54 112 43 56 70	91	3.39E+05	1.38E+06
B	1		3: 82 56 71	95	5.55E+05	2.26E+06
B	3		4: 83 71 57 41	92	1.23E+05	5.01E+05
B	4			66	2.31E+04	9.39E+04
+Methyl vinyl ketone	78-94-4	13.82		Sweet			A	6		4: 70 55 39 82	67	1.34E+04	
Tranylcypromine	155-09-9	13.91					A	2		7: 132 117 102 118 91 115 99	69	1.31E+06	
+Propanoic acid	79-09-4	13.91	Pungent, Rancid, Soy	Pungent, Acidic, Cheesy, Vinegar		3.55E-02	A	6		3: 73 74 60	65	1.76E+05	4.97E+06
5-methylindane	874-35-1	13.91					A	1			88	2.94E+05	
A	2		5: 132 116 39 131 57	90	1.91E+06	
B	1		5: 91 132 115 116 64	78	4.44E+04	
C	1		5: 132 115 131 65 91	75	1.10E+05	
2-ethenyl-1,3-dimethylbenzene	2039-90-9	13.91					A	1			92	2.94E+05	
A	2		7: 132 117 102 118 91 115 99	94	1.31E+06	
B	1		5: 91 132 115 116 64	82	4.44E+04	
C	1		5: 132 115 131 65 91	82	1.10E+05	
Propylene glycol	57-55-6	13.98					A	1		3: 45 43 41	72	1.50E+05	
A	2			68	1.20E+05	
A	3			67	7.50E+04	
A	7		6: 45 46 39 42 41 47	71	3.01E+05	
B	3			72	4.64E+04	
B	4		2: 42 43	67	7.41E+04	
C	1		4: 44 90 38 37	69	6.13E+05	
C	2		17: 45 43 47 44 55 90 76 53 73 115 71 41 60 56 51 54 40	69	6.30E+06	
Indane	496-11-7	13.98					A	1		3: 118 115 117	68	1.10E+05	
2-chloroacetophenone	532-27-4	14.09		Apple blossom		2.57E-02	A	5		6: 105 51 77 52 78 63	78	6.57E+05	2.56E+07
A	7			76	1.00E+06	3.89E+07
B	1		3: 77 78 50	76	3.34E+05	1.30E+07
B	2			71	2.25E+04	8.76E+05
B	3		3: 105 106 107	71	1.30E+05	5.06E+06
B	4		6: 105 77 106 78 51 107	74	1.07E+05	4.17E+06
+Benzaldehyde	100-52-7	14.09	Almond, Burnt sugar	Fruity	3.00E-03	4.17E-02	A	5			98	8.98E+05	2.15E+07
A	7			98	1.00E+06	2.40E+07
B	1		3: 77 78 50	97	5.17E+05	1.24E+07
B	2		3: 106 105 77	83	4.03E+04	9.66E+05
B	3		3: 105 106 107	89	1.30E+05	3.12E+06
B	4		6: 105 77 106 78 51 107	93	1.07E+05	2.57E+06
+Ethyl lactate	97-64-3	14.10	Fruit	Sharp, Tart, Fruity, Buttery, Butterscotch		1.62E+00	A	6		4: 45 46 75 47	87	2.84E+05	1.75E+05
B	3		4: 43 207 42 46	84	5.88E+04	3.63E+04
C	1		10: 72 90 56 73 37 60 74 48 71 76	80	9.99E+06	6.16E+06
C	2		12: 72 39 56 73 41 71 60 53 49 52 48 40	80	6.67E+06	4.11E+06
C	3		13: 55 42 58 41 60 56 38 73 91 54 89 74 132	79	1.64E+07	1.01E+07
Isobutyrophenone	611-70-1	14.10		Green			B	1		3: 77 78 50	69	3.34E+05	
Dimethyl octanol	106-21-8	14.11		Waxy, Soapy, Aldehydic, Leathery, Musty, Citrus, Green			C	1		8: 54 70 111 67 97 56 53 110	82	7.44E+05	
C	2		6: 70 41 57 79 84 97	66	2.13E+05	
1-Dodecanol	112-53-8	14.11	Fat, Wax	Earthy, Soapy, Waxy, Fatty, Honey, Coconut		1.26E-02	C	1			93	2.64E+05	2.10E+07
C	2		6: 70 41 57 79 84 97	76	1.91E+05	1.52E+07
+1-Decanol	112-30-1	14.11	Fat	Fatty, Waxy, Floral, Orange, Sweet, Clean, Watery		1.82E-02	C	1			75	6.38E+04	3.51E+06
C	2		6: 70 41 57 79 84 97	86	7.90E+04	4.34E+06
1-Nonanol	143-08-8	14.12	Fat, Green	Fresh, Clean, Fatty, Floral, Rose, Orange, Dusty, Wet, Oily	5.00E+01	2.24E-03	C	1			76	3.03E+04	1.35E+07
C	2			71	7.50E+04	3.35E+07
+Undecane	1120-21-4	14.13	Alkane			1.17E+00	C	1		7: 85 99 71 110 98 68 39	68	2.43E+05	2.07E+05
+Nonane	111-84-2	14.13	Alkane	Gasoline		1.26E+00	C	1		7: 85 99 71 110 98 68 39	77	2.17E+05	1.72E+05
+Dodecane	112-40-3	14.13	Alkane	Alkane		2.04E+00	C	1		7: 85 99 71 110 98 68 39	77	2.17E+05	1.06E+05
+Tridecane	629-50-5	14.14	Alkane	Alkane		2.14E+00	A	4			68	1.59E+04	7.44E+03
C	1		7: 85 99 71 110 98 68 39	68	2.43E+05	1.14E+05
2,2-dimethylbutane	75-83-2	14.15					A	4			82	1.59E+04	
3-isopropyl phenol	618-45-1	14.19					A	1		7: 121 77 55 136 67 120 79	77	3.82E+05	
A	2		7: 122 105 103 93 121 51 57	82	7.66E+05	
C	3		20: 121 136 122 103 78 77 105 80 41 106 107 39 43 94 120 115 52 135 67 54	81	1.39E+06	
3-(1-methylethyl)-phenol methylcarbamate	64-00-6	14.20					A	1		8: 121 105 136 106 91 77 79 265	76	7.43E+05	
A	2		13: 105 121 51 79 136 77 78 53 103 106 39 120 43	74	7.94E+05	
A	4		2: 136 121	65	6.07E+04	
A	7		13: 121 78 136 68 103 117 80 52 51 77 106 107 81	66	3.18E+05	
B	1		3: 136 91 107	74	4.44E+04	
Acetone cyanohydrin	75-86-5	14.27					B	1		4: 70 83 112 69	69	3.20E+04	
B	3		1: 70	70	8.97E+03	
1,4-diethylbenzene	105-05-5	14.47					A	1			87	9.45E+04	
A	2		9: 120 55 115 93 135 52 108 103 133	85	5.16E+05	
o-cymene	527-84-4	14.47				7.94E-04	A	2		18: 91 52 119 106 134 93 55 105 92 115 103 117 79 65 120 63 133 116	88	4.83E+05	6.08E+08
1,2-diethylbenzene	135-01-3	14.47					A	1			85	9.45E+04	
A	2		18: 91 52 119 106 134 93 55 105 92 115 103 117 79 65 120 63 133 116	84	8.70E+05	
p-tert-butylphenol	98-54-4	14.48		Leathery			A	7			68	1.54E+04	
tert-butyl-benzene	98-06-6	14.48					A	1			89	6.59E+04	
A	2			86	3.38E+05	
B	1		3: 91 120 134	76	6.51E+04	
o-methylacetophenone	577-16-2	14.48		Floral		6.61E-03	A	1			89	6.59E+04	9.98E+06
2-methoxyethanol	109-86-4	14.62					A	6		2: 43 55	65	1.39E+05	
+2-Butanol	78-92-2	14.66	Wine	Sweet, Apricot	1.70E+00		C	3		13: 45 43 47 44 55 46 42 54 60 58 76 38 86	69	2.96E+07	1.74E+07
Maltol	118-71-8	14.67	Caramel	Sweet, Caramel, Cotton candy, Jam, Fruity, Baked bread			A	4		3: 98 126 71	66	5.65E+03	
Linalyl acetate	115-95-7	15.09	Sweet, Fruit	Herbal		8.91E-03	A	4			77	2.39E+04	2.68E+06
A	6		1: 83	74	4.56E+04	5.12E+06
Geranyl butyrate	106-29-6	15.09	Fruit, Rose, Apple	Sweet, Fruity, Rose, Waxy Raspberry, Tropical			A	4			68	1.45E+04	
Isobornyl thiocyanoacetate	115-31-1	15.11					A	1		20: 92 105 80 51 117 66 137 122 62 81 64 54 104 63 76 108 103 134 38 43	66	3.33E+07	
A	2		20: 68 93 67 94 136 59 107 91 53 39 81 95 105 55 65 66 119 137 52 96	66	2.21E+07	
A	3		9: 81 137 95 106 122 43 42 108 103	67	9.69E+05	
A	6		20: 55 65 77 93 39 41 136 80 43 81 121 86 139 97 53 94 91 52 105 84	70	7.74E+05	
A	7		20: 93 69 80 71 72 122 41 92 55 107 136 65 94 53 81 105 45 56 96 82	68	3.68E+05	
B	3		10: 72 139 94 65 70 57 67 92 52 54	73	1.18E+06	
Linalyl propionate	144-39-8	15.11		Fresh, Bergamot, Lily, Woody, Rose, Rum			B	3		17: 121 93 41 82 80 94 70 67 105 68 84 51 56 53 72 137 126	67	6.45E+05	
Linalool	78-70-6	15.12	Flower, Lavender	Floral	6.00E-03	5.37E-02	A	1		3: 69 71 43	85	9.62E+04	1.79E+06
A	2			89	1.00E+05	1.87E+06
A	5			91	3.31E+05	6.16E+06
A	6			96	8.95E+05	1.67E+07
A	7		20: 93 69 80 71 72 122 41 92 55 107 136 65 94 53 81 105 45 56 96 82	95	3.68E+05	6.85E+06
B	3		10: 72 139 94 65 70 57 67 92 52 54	97	1.18E+06	2.19E+07
Ethyl cyclohexane	1678-91-7	15.17					C	1		6: 55 83 84 67 169 139	71	3.95E+05	
1-methyl-1H-imidazole	616-47-7	15.20					C	3		3: 82 69 168	67	1.48E+05	
cis-2-pinanol	4948-29-2	15.41		Herbal			A	5			72	1.30E+04	
A	6		20: 81 99 79 97 121 67 77 43 68 83 95 71 86 72 94 108 107 69 57 105	95	8.31E+05	
A	7			79	1.66E+04	
B	3		4: 94 93 58 72	92	5.15E+05	
trans-carveol	1197-07-5	15.51	Caraway, Solvent	Caraway, Solvent, Spearmint			C	1		19: 109 106 43 137 67 119 69 39 134 65 79 94 110 41 82 105 117 115 121	74	1.36E+06	
β-cyclocitral	432-25-7	15.52	Mint	Tropical, Saffron, Herbal, clean, Rose, Sweet, Tobacco, Damascenone, Fruity			C	1			87	1.15E+06	
C	2		20: 95 134 119 138 77 106 121 152 137 67 107 41 79 65 91 78 117 120 110 55	87	9.49E+05	
C	3			86	8.54E+05	
tetrahydro-2-methyl-2-furanol	7326-46-7	15.57					C	1		7: 71 43 72 78 39 41 82	76	2.27E+05	
Fenchyl alcohol	1632-73-1	15.72	Camphor	Camphor, Borneol, Pine, Woody, Dry, Sweet, Lemon			A	1			76	4.88E+04	
A	2		2: 80 81	67	7.96E+04	
A	4		12: 81 107 43 41 83 72 71 69 121 53 96 67	92	2.37E+05	
A	5			78	5.65E+04	
A	6		20: 81 107 72 84 41 69 55 111 92 71 93 123 121 83 122 57 43 95 79 77	98	1.84E+06	
A	7			72	1.94E+04	
B	3			99	2.28E+06	
B	4			83	2.67E+04	
+1-methyl-1H-pyrrole	96-54-8	15.72		Smoky, Woody, Herbal			A	2		3: 81 80 69	69	2.65E+04	
(-)-terpinen-4-ol	20126-76-5	16.20					A	6			70	1.45E+04	
1-terpinen-4-ol	562-74-3	16.20	Turpentine, Nutmeg, Must	Woody, Ceding, mentholic, Citrus, Terpiny, Spicy			A	6			74	1.45E+04	
Thujone	546-80-5	16.22		Cedar leaf		1.29E-01	C	3			72	8.49E+03	6.59E+04
2-Methyl-4-(1-methylethyl)-2-cyclohexenone	41469-46-9	16.33					C	1		4: 109 81 95 65	77	5.92E+04	
C	2			80	3.89E+04	
C	3			82	3.07E+04	
Camphor	76-22-2	16.33	Camphor	Camphorous	5.13E-02		C	2			69	3.89E+04	7.58E+05
C	3			69	6.43E+04	1.25E+06
Pulegone	89-82-7	16.34		Peppermint, Camphor, Fresh, Herbal, Buchu	3.39E-03		C	1		4: 152 67 109 81	71	6.25E+04	1.85E+07
C	2			76	3.90E+04	1.15E+07
2,2,4-trimethylpentane	540-84-1	16.57					A	4		6: 57 99 56 140 183 86	77	3.93E+05	
γ-hexalactone	695-06-7	17.20	Coumarin, Sweet	Tonka			B	3		1: 85	78	3.62E+04	
Borneol	507-70-0	17.60	Camphor	Pine, Woody, Camphor		2.09E-03	A	6		6: 139 77 110 92 94 91	96	8.39E+05	4.01E+08
B	3			98	7.46E+05	3.57E+08
Isobornyl acetate	125-12-2	17.60		Balsamic			A	6		6: 139 77 110 92 94 91	77	8.39E+05	
B	3			79	7.46E+05	
+Laevo-borneol	464-45-9	17.60		Pine, Woody, Camphor			A	6			95	4.15E+05	
B	3		19: 95 69 121 79 105 140 55 67 92 68 43 110 70 91 111 108 42 57 113	98	6.63E+05	
+α-terpineol	98-55-5	17.73	Oil, Anise, Mint	Floral		3.72E-02	A	1			84	6.15E+04	1.66E+06
A	2			80	3.85E+04	1.04E+06
A	4			83	3.03E+04	8.17E+05
A	5			80	3.75E+04	1.01E+06
A	6			94	7.72E+05	2.08E+07
A	7			80	2.54E+04	6.84E+05
B	3			95	5.95E+05	1.60E+07
α-terpinyl acetate	80-26-2	17.73	Wax	Herbal, Bergamot, Lavender, Lime, Citrus			A	3		20: 121 136 68 93 41 77 94 51 52 54 78 43 95 80 69 65 42 119 63 103	67	1.20E+06	
A	4		2: 136 121	68	4.41E+04	
A	5		7: 136 80 93 95 41 43 81	77	6.84E+04	
B	1		6: 136 92 63 119 80 66	69	4.56E+05	
Terpinyl butyrate	2153-28-8	17.74		Sour, Rosemary, Fruity, Balsam			A	7		4: 136 94 68 93	68	1.57E+04	
2-ethyl-3,5-dimethylpyridine	1123-96-2	17.91					A	1		4: 68 82 134 133	66	1.28E+05	
B	1		2: 134 135	74	2.22E+04	
+p-cresyl acetate	140-39-6	18.14		Narcissus, Phenolic, Animal		7.76E-04	A	7		1: 108	65	9.23E+03	1.19E+07
m-tert-butylphenol	585-34-2	18.15					A	7		5: 135 80 108 79 91	68	6.76E+04	
Verbenone	80-57-9	18.16		Camphor, Menthol, Celery			A	5			75	9.46E+03	
A	7			82	2.55E+04	
1-Tetradecanol	112-72-1	18.32	Coconut	Fruity, Waxy, Orris, Coconut			C	1			80	8.39E+04	
3-methylhexane	589-34-4	18.32					A	4			72	1.66E+04	
C	1		10: 57 98 82 71 68 43 67 56 70 127	75	3.62E+04	
+1-Tridecene	2437-56-1	18.33					C	2			80	4.71E+04	
1-undecanol	112-42-5	18.34	Mandarin	Waxy		6.76E-02	C	1			74	6.38E+04	9.44E+05
C	2		6: 70 41 57 79 84 97	82	7.90E+04	1.17E+06
Octyl formate	112-32-3	18.34		Fruity, rose, Orange, Waxy, Cucumber			C	2			67	1.25E+04	
α-copaene	3856-25-5	18.39	Wood, Spice	Wood			A	1			71	3.09E+04	
α-cubebene	17699-14-8	18.50	Herb, Wax	Herb			A	1			73	1.57E+04	
(+)-sativene	3650-28-0	19.40					A	1			78	5.89E+04	
A	5		20: 93 69 120 148 106 68 55 92 189 95 149 175 135 162 190 136 83 91 53 103	75	2.37E+07	
Nitro cyclohexane	1122-60-7	19.46					C	2		2: 83 55	67	2.04E+04	
β-caryophyllene	87-44-5	19.66	Wood, Spice	Spice	6.40E-02		A	1		20: 133 69 79 161 105 120 136 81 77 106 119 162 121 39 109 94 175 92 82 123	100	6.01E+06	9.40E+07
A	2		17: 189 106 92 41 148 190 81 80 93 78 95 121 77 161 94 91 120	99	2.39E+06	3.73E+07
A	3			77	3.32E+04	5.19E+05
A	4			80	3.35E+04	5.24E+05
A	6		20: 41 133 93 69 107 147 148 120 66 55 121 80 42 176 119 95 53 43 145 136	99	4.19E+06	6.55E+07
A	7		10: 134 124 96 66 112 190 122 110 177 138	89	1.05E+06	1.64E+07
B	3			99	3.11E+06	4.86E+07
B	4		14: 94 69 120 135 107 163 80 78 134 176 161 109 63 82	99	1.37E+06	2.14E+07
+Benzyl alcohol	100-51-6	19.74	Sweet, Flower	Floral			A	5		11: 78 53 109 149 39 129 66 65 123 134 202	96	2.16E+07	
A	7			96	5.83E+06	
B	1		13: 108 79 78 51 91 109 90 39 86 62 92 74 37	100	3.59E+06	
B	2		11: 108 107 77 80 76 106 49 91 105 53 41	99	8.86E+05	
Tyramine	51-67-2	19.74		Meaty			A	5		6: 51 85 38 62 90 75	70	9.20E+06	
A	7		5: 90 62 109 37 61	70	3.71E+06	
B	1		1: 53	70	4.00E+06	
B	2			72	6.78E+05	
α-guaiene	3691-12-1	19.85	Wood, Balsamic	Wood			A	1			80	2.88E+05	
A	2		13: 106 189 133 123 162 93 204 95 120 108 94 205 105	91	5.50E+05	
A	5			72	3.36E+05	
A	6		16: 107 147 108 93 94 106 91 67 105 189 121 81 51 69 119 53	94	4.50E+05	
B	3			94	3.21E+05	
B	4		13: 107 204 135 79 133 119 105 147 81 148 73 65 95	88	9.51E+04	
+Dimethylsulfone	67-71-0	20.12	Sulfur, Burnt	Sulfurous, Burnt			A	7		2: 94 79	80	1.93E+04	
δ-cadinene	483-76-1	20.20	Thyme, Medicine, Wood	Herbal			A	1		20: 161 204 190 122 39 202 107 55 65 134 41 159 69 81 149 67 109 53 78 117	66	4.74E+05	
A	5			74	1.51E+04	
2,6-pyridinediamine	141-86-6	20.49					A	5		1: 109	71	4.03E+03	
α-humulene	6753-98-6	20.53	Wood	Wood	1.20E-01		A	1		19: 147 93 121 67 92 105 81 109 39 80 91 119 77 57 41 43 135 103 120	97	1.68E+06	1.40E+07
A	2			91	1.76E+05	1.47E+06
A	5		20: 93 80 121 107 79 92 147 91 70 41 105 109 205 94 122 189 106 82 204 95	98	3.99E+06	3.32E+07
A	6		20: 107 105 80 67 190 109 94 95 106 147 92 41 68 83 189 108 65 52 42 205	96	1.55E+06	1.29E+07
A	7		19: 92 79 94 105 95 91 148 63 65 204 120 41 123 82 135 78 66 39 128	97	1.42E+06	1.19E+07
B	3		20: 121 93 107 148 91 106 123 66 39 122 42 175 204 95 205 40 73 120 133 129	97	1.47E+06	1.23E+07
B	4			93	2.10E+05	1.75E+06
β-selinene	17066-67-0	21.25	Herb	Herb			A	1			93	3.35E+05	
A	2			86	6.14E+04	
A	6		14: 161 135 108 119 163 81 94 109 105 78 41 93 82 149	92	3.26E+05	
A	7		15: 161 162 134 94 190 43 91 81 204 121 123 95 92 131 175	72	1.56E+05	
B	3			92	1.84E+05	
B	4			85	4.15E+04	
Longifolene	475-20-7	21.27		Wood			A	1		20: 133 69 79 161 105 120 136 81 77 106 119 162 121 39 109 94 175 92 82 123	89	6.01E+06	
A	2		17: 189 106 92 41 148 190 81 80 93 78 95 121 77 161 94 91 120	89	2.39E+06	
A	5		17: 147 205 68 133 161 148 189 105 175 93 107 135 109 123 53 69 134	91	6.66E+05	
A	6		20: 41 133 93 69 107 147 148 120 66 55 121 80 42 176 119 95 53 43 145 136	90	4.19E+06	
A	7			87	1.16E+05	
B	3			90	3.11E+06	
B	4		14: 94 69 120 135 107 163 80 78 134 176 161 109 63 82	88	1.37E+06	
Alloaromadendrene	25246-27-9	21.41	Wood	Wood			B	3		20: 55 135 96 121 79 93 105 161 148 106 204 120 91 80 127 94 77 122 205 104	71	1.21E+06	
α-bulnescene	3691-11-0	21.41					A	2			89	1.96E+05	
A	6		6: 105 136 69 43 42 109	94	1.08E+06	
B	3		20: 55 135 96 121 79 93 105 161 148 106 204 120 91 80 127 94 77 122 205 104	96	1.07E+06	
B	4			92	4.36E+05	
α-gurjunene	489-40-7	21.43	Wood, Balsamic	Wood			A	1			85	2.68E+05	
A	2			73	1.22E+04	
A	5			88	2.34E+05	
A	6		3: 145 147 109	82	2.85E+05	
A	7		10: 147 131 107 133 109 204 119 79 95 105	81	1.34E+05	
B	3			81	5.86E+04	
B	4		9: 106 119 51 149 162 161 123 81 117	81	3.10E+04	
Aromadendrene	489-39-4	21.48	Wood	Wood			A	1			65	6.94E+04	
A	2		8: 82 93 147 121 162 67 65 133	73	1.48E+05	
A	5			71	8.73E+04	
A	6			81	5.80E+04	
A	7			91	1.16E+05	
B	3		19: 161 147 105 129 133 204 106 109 95 77 145 82 92 91 108 190 120 41 117	87	9.66E+04	
2,4,6-trimethylpyridine	108-75-8	21.66					A	2		4: 121 67 39 106	65	1.24E+05	
A	6			72	1.01E+04	
+Phenol	108-95-2	21.68	Phenolic	Phenolic		1.10E-01	B	1			78	1.73E+04	1.58E+05
Dyclocaine	586-60-7	21.69					A	1			70	6.94E+04	
(-)-Aristolene	6831-16-9	21.74					A	1		15: 108 119 79 135 204 189 133 187 106 148 67 55 42 43 78	74	4.92E+05	
A	6			81	5.14E+04	
B	3		11: 161 204 79 148 107 53 109 81 202 108 105	83	8.64E+04	
+2-ethylphenol	90-00-6	21.91		Phenolic			A	5		2: 122 107	71	3.34E+03	
(+)-calarene	17334-55-3	22.08					A	1		20: 121 91 107 162 95 105 189 81 136 135 134 79 39 110 92 57 190 53 160 146	73	2.22E+06	
A	2			71	2.89E+04	
A	5		17: 147 109 161 91 148 204 135 133 92 189 107 94 93 159 134 41 149	78	6.42E+05	
A	6		17: 161 121 122 149 136 67 189 55 135 81 145 162 148 39 80 41 134	70	8.30E+05	
B	3		16: 148 105 161 162 205 92 67 133 107 79 135 115 134 120 93 119	78	1.44E+05	
B	4		20: 77 147 161 67 134 189 65 121 105 133 82 95 55 79 120 109 43 83 108 78	73	6.04E+05	
α-cedrene	469-61-4	22.08		Woody, Cedar, Sweet, Fresh			A	5		15: 119 204 161 93 65 69 133 80 121 135 134 41 189 94 79	72	4.47E+05	
A	7			72	1.74E+04	
Longicyclene	1137-12-8	22.10					A	5		17: 109 93 189 190 80 131 133 204 55 121 115 79 105 145 82 107 135	78	1.57E+05	
B	4		7: 134 189 81 204 161 106 78	72	3.50E+05	
γ-gurjunene	22567-17-5	22.14		Musty			A	1		20: 121 91 107 162 95 105 189 81 136 135 134 79 39 110 92 57 190 53 160 146	92	2.49E+06	
A	2			84	6.30E+04	
A	5		20: 93 147 77 105 129 108 79 189 119 81 91 135 106 175 131 145 205 51 95 109	92	4.50E+06	
A	6		18: 161 204 108 105 205 107 122 81 55 53 109 148 39 92 79 77 162 106	89	9.02E+05	
A	7			89	5.08E+05	
B	3		20: 161 122 107 149 204 105 109 205 95 65 79 55 135 134 77 141 91 41 92 108	91	7.69E+05	
B	4		20: 148 79 161 107 95 145 204 67 93 120 105 122 41 91 106 162 205 108 39 150	90	4.49E+05	
α-longipinene	5989-08-2	22.18					A	6		20: 93 121 122 204 133 115 119 135 91 205 117 105 77 159 176 54 95 162 51 163	70	1.01E+06	
Cedryl acetate	77-54-3	22.18		Wood			B	4		17: 119 105 204 69 55 190 149 107 67 96 95 205 175 106 187 147 109	65	2.31E+05	
Valencene	4630-07-3	22.19	Green, Oil	Citrus			A	1		20: 133 121 161 92 204 79 107 91 119 52 190 81 93 55 78 53 115 131 206 129	96	3.06E+06	
A	2			88	1.47E+05	
A	5		20: 161 204 131 133 91 53 106 190 68 108 43 66 77 94 162 78 148 73 160 143	96	3.46E+06	
A	6		16: 134 78 135 108 204 147 161 39 82 95 79 119 107 175 52 131	95	1.39E+06	
A	7			95	8.47E+05	
B	3		20: 161 91 204 133 145 78 135 81 134 79 55 119 120 63 93 53 107 108 174 122	96	1.14E+06	
B	4		20: 77 147 161 67 134 189 65 121 105 133 82 95 55 79 120 109 43 83 108 78	94	4.66E+05	
2-hydroxyethyl acrylate	5951-61-1	22.61					A	1			79	2.64E+04	
A	2			77	2.03E+04	
A	5			82	6.28E+04	
A	6		4: 133 119 109 161	79	5.29E+04	
A	7		13: 107 121 149 81 79 42 189 190 82 161 39 136 97	73	2.79E+05	
B	3			80	4.60E+04	
B	4		20: 148 79 161 107 95 145 204 67 93 120 105 122 41 91 106 162 205 108 39 150	77	7.39E+05	
+Butylated Hydroxytoluene	128-37-0	22.66		Mild, Phenolic, Camphor			B	1			84	3.39E+04	
B	2			90	7.76E+04	
Xylazine	7361-61-7	22.67					B	2			66	7.76E+04	
2,3,6-trimethylpyridine	1462-84-6	23.95					A	2		5: 41 134 120 121 83	65	1.78E+04	
Toluene-2,4-diamine	95-80-7	23.97					A	2		4: 122 121 105 96	70	1.18E+04	
Propofol	2078-54-8	23.97		Phenolic			A	1		20: 93 164 108 107 178 80 124 135 79 106 122 145 41 120 55 94 91 103 95 149	68	7.95E+05	
A	2			70	1.43E+05	
1-(3,6-Dimethyl-2-pyrazinyl)-2-methyl-1-propanone	145984-66-3	23.98					A	2		14: 108 123 93 67 163 81 178 107 105 91 79 66 55 145	68	1.38E+05	
Methyl isoeugenol	93-16-3	23.98	Clove, Spice	Spice			A	1		14: 163 41 93 108 107 105 119 115 91 149 95 145 78 160	67	1.50E+06	
A	2			66	1.43E+05	
Caryophyllene oxide	1139-30-6	24.09	Herb, Sweet, Spice	Woody			A	5			74	2.69E+04	
p-acetanisole	100-06-1	24.58		Anisic			C	1		2: 135 150	68	1.31E+04	
C	3			69	6.11E+03	
3-methyl-5-(1-methylethyl)-Phenol methylcarbamate	2631-37-0	24.64					C	1		2: 135 150	70	1.31E+04	
C	2			84	2.37E+04	
C	3		1: 150	73	2.09E+04	
												
Thymol	89-83-8	24.78		Herbal		1.55E-02	C	1			91	4.48E+04	2.89E+06
C	2			82	2.37E+04	1.53E+06
C	3			70	6.20E+03	4.00E+05
+Carvacrol	499-75-2	24.78		Spicy		1.12E-02	C	1			92	4.48E+04	3.99E+06
							C	2			84	2.37E+04	2.11E+06
C	3			72	6.20E+03	5.53E+05
2,4-di-tert-butylphenol	96-76-4	26.36		Phenolic			A	4		1: 191	68	2.90E+04	
α-bisabolol	72691-24-8	26.43					A	5			77	3.95E+04	
Cyclobarbital	52-31-3	35.80					A	7		1: 207	65	9.20E+03	
1,4-Dioxane	123-91-1	38.37				5.50E+00	B	3		2: 58 88	71	2.39E+03	4.34E+02

If two references of ODTs are available, ODT from Devos, et al. [5] is used to calculate OAV. RT = Retention Time. ODT = Odor Detection Threshold. Code, see Section 1.5. Models = significant ions used for identification/semi-quantitation, # before colon is number of significant ions, #’s after colon are m/z. Net % match as calculated using AMDIS and target specialty mass spectral libraries. PAC = Peak Area Counts, and refers to relative abundance as given by the mass spectral detector. OAV = Odor Activity Value, and is calculated as ratio of PAC: OAV. Underlined items highlight the compounds found in Pseudo Scent Marijuana. + Compounds indicate confirmation with reference standards, matching retention time and spectra.

**Table 4:** Summary of VOCs emitted from all illicit cocaine samples (sample code D in Section 1.5) and Sigma Pseudo™ Narcotic Scent Cocaine formulation (sample code E in Section 1.5) and sampled over 1 h at room temperature. **Sigma Pseudo™ Narcotic Scent Cocaine formulation is indicated by underlined fonts.**

**Compound**	**CAS**	**RT (min)**	**Published descriptors**			**Published ODT (ppm)**		**Sample code**		**Models**	**Net % match**	**PAC**	**OAV**
			**Flavornet**[7]	**TGSC**[8]		**LRI & Odor**[6]	**Devos et al.**[5]						
Ethylene oxide	75-21-8	1.07					8.51E+02	D	4		66	3.83E+06	4.50E+03
E	1		66	2.28E+06	2.68E+03
+2-nitropropane	79-46-9	1.11					7.24E+00	D	2	4: 41 43 56 39	88	2.92E+04	4.03E+03
D	4		73	3.05E+04	4.21E+03
D	5	3: 39 43 41	83	4.84E+03	6.69E+02
2,4-dimethylpentane	108-08-7	1.16					8.71E+01	D	1	4: 57 85 43 99	70	1.41E+05	1.62E+03
D	3	5: 53 100 70 86 57	83	8.33E+05	9.57E+03
D	4	4: 43 56 42 84	69	1.90E+04	2.18E+02
1,2-dimethyl hydrazine	540-73-8	1.18						D	1	1: 45	74	1.91E+04	
Ethylenimine	151-56-4	1.20						D	2	4: 43 42 56 41	68	7.08E+04	
Isobutane	75-28-5	1.24					1.00E+01	D	1	6: 43 42 41 57 72 39	83	1.26E+06	1.26E+05
D	2	9: 43 42 41 57 39 55 56 53 58	84	1.43E+06	1.43E+05
D	3	11: 43 42 41 57 72 56 55 39 38 71 51	85	3.13E+06	3.13E+05
D	4	4: 43 42 41 72	82	2.27E+05	2.27E+04
D	5	7: 41 43 42 39 72 57 55	81	1.53E+05	1.53E+04
Ethyl Chloride	75-00-3	1.26						D	1	2: 64 66	75	1.37E+04	
+Butane	106-97-8	1.26					2.04E+02	D	1	6: 43 58 42 41 37 45	82	4.20E+06	2.06E+04
D	2	4: 41 43 56 39	79	2.92E+04	1.43E+02
D	4		91	3.05E+04	1.49E+02
D	5	3: 43 56 58	87	5.11E+04	2.50E+02
Trichloromonofluoromethane	75-69-4	1.27						D	2	2: 103 101	77	5.39E+03	
+Acetaldehyde	75-07-0	1.28	Pungent, Ether	Pungent, Ethereal, Aldehydic, Fruity		1.50E-02	1.86E-01	D	1	2: 44 43	81	3.01E+04	1.61E+05
D	2	1: 44	81	2.68E+04	1.44E+05
D	4	3: 44 43 42	91	6.31E+04	3.39E+05
D	5		68	2.60E+03	1.40E+04
+Ethyl ether	60-29-7	1.31		Ethereal				D	4	2: 59 45	86	1.43E+04	
Isoprene	78-79-5	1.33						D	4	2: 53 67	82	2.29E+04	
4-methyldecane	2847-72-5	1.39						D	1	4: 56 57 55 43	72	7.44E+04	
D	2	8: 43 71 70 41 86 55 57 56	65	5.04E+05	
D	3	13: 70 56 71 113 99 85 41 69 67 42 72 44 114	84	8.36E+05	
D	4	2: 42 70	65	2.98E+05	
2-methylpentane	107-83-5	1.39						D	1	2: 57 70	97	3.19E+05	
D	2	8: 43 71 70 41 86 55 57 56	98	5.04E+05	
D	3	6: 43 71 42 39 55 56	97	5.78E+05	
D	4	2: 42 70	96	2.98E+05	
D	5	6: 41 43 71 70 55 39	96	2.07E+05	
2,3-dimethylbutane	79-29-8	1.40						D	3	3: 42 41 43	65	2.60E+05	
D	4	9: 43 71 41 39 55 86 42 53 72	87	2.93E+05	
Hexane	110-54-3	1.44	Alkane				2.19E+01	D	1	2: 43 42	96	1.06E+05	4.83E+03
D	2	4: 43 42 56 41	83	7.76E+04	3.55E+03
D	3	9: 57 43 41 56 86 39 58 55 70	99	2.29E+06	1.05E+05
D	4		90	4.09E+05	1.87E+04
D	5	4: 57 56 41 86	90	3.60E+05	1.64E+04
Cyclopentane	287-92-3	1.45		Petroleum				D	4	3: 55 70 53	77	3.85E+04	
D	5	2: 42 55	83	2.96E+04	
2-methylaziridine	75-55-8	1.45						D	1	3: 56 41 57	80	7.68E+04	
D	2		81	7.80E+04	
D	3	1: 57	79	1.35E+05	
D	4		81	6.54E+04	
D	5		82	5.43E+04	
3-methylpentane	96-14-0	1.45						D	1	3: 56 41 57	87	7.68E+04	
D	2		93	7.80E+04	
D	3	1: 57	87	1.35E+05	
D	4		96	6.37E+04	
D	5	2: 56 55	92	1.57E+05	
E	1	2: 56 57	67	3.48E+03	
Isocyanatomethane	624-83-9	1.46						D	1	4: 56 57 55 43	66	8.44E+04	
D	3	5: 57 112 85 43 113	80	8.15E+04	
D	5	2: 56 55	80	1.23E+05	
E	1	2: 56 57	79	3.48E+03	
2-hydroxy propanenitrile	78-97-7	1.48						D	3	9: 43 56 42 53 55 87 54 85 50	73	8.86E+05	
D	5	2: 56 55	65	8.32E+04	
3,4,5-trimethyl-1-hexene	56728-10-0	1.51						D	1	9: 43 71 42 56 41 70 57 39 38	67	3.11E+05	
D	2	8: 43 71 70 41 86 55 57 56	67	5.04E+05	
D	3		69	4.99E+05	
D	4	2: 42 70	68	2.98E+05	
D	5	3: 43 57 71	68	1.72E+05	
+Propanal	123-38-6	1.57	Solvent, Pungent	Earthy, Alcohol, Wine, Whiskey, Cocoa, Nutty		1.00E-02	2.69E-02	D	1	3: 58 57 41	68	1.47E+04	5.46E+05
+Acetic anhydride	108-24-7	1.62		Sharp, Vinegar			5.89E-01	D	1	2: 61 43	69	2.06E+05	3.50E+05
D	3	1: 43	81	3.68E+04	6.25E+04
D	4	3: 43 58 42	81	3.40E+04	5.77E+04
D	5	2: 43 42	73	4.76E+03	8.08E+03
E	1	1: 43	65	1.08E+03	1.83E+03
2,2,4,4-tetramethyl-3-pentanone	815-24-7	1.65						D	1	4: 57 85 43 99	68	1.41E+05	
D	3	5: 112 57 85 41 55	75	1.28E+05	
2-methyl-2-propanamine	75-64-9	1.65						D	4	4: 41 57 37 85	91	1.22E+05	
2,2,4,4-tetramethyl-3-pentanone	815-24-7	1.65						D	4	4: 41 57 37 85	73	3.53E+04	
+Acetone	67-64-1	1.66		Solvent			1.45E+01	D	1	6: 43 58 42 41 37 45	99	4.20E+06	2.91E+05
D	2	2: 43 58	88	1.55E+05	1.07E+04
D	3	3: 58 43 42	88	9.15E+04	6.33E+03
D	4	3: 43 58 39	97	9.33E+05	6.46E+04
D	5	6: 43 58 57 42 37 44	97	5.36E+05	3.71E+04
E	1	2: 58 43	81	1.03E+04	7.11E+02
+Methyl acetate	79-20-9	1.68		Ethereal				D	1	1: 43	85	1.36E+05	
D	4	3: 43 74 39	95	1.69E+05	
D	5	3: 74 43 42	95	1.98E+05	
+Acrolein	107-02-8	1.71		Almond, Cherry			1.74E-01	D	3	1: 56	66	6.09E+04	3.50E+05
+Propene	115-07-1	1.71					5.25E+01	D	3	3: 41 39 42	77	3.28E+04	6.25E+02
Methacrylic anhydride	760-93-0	1.71						D	3	3: 41 39 42	75	2.28E+04	
Isobutyraldehyde	78-84-2	1.76	Pungent, Malt, Green	Spicy			4.07E-02	D	1		91	4.35E+04	1.07E+06
D	2	9: 43 42 41 57 39 55 56 53 58	77	1.43E+06	3.50E+07
D	3	11: 43 42 41 57 72 56 55 39 38 71 51	78	3.13E+06	7.68E+07
D	4	4: 43 42 41 72	76	2.27E+05	5.57E+06
D	5	7: 41 43 42 39 72 57 55	75	1.53E+05	3.77E+06
1-(ethenyloxy)-butane	111-34-2	1.89						D	3	5: 53 100 70 86 57	69	6.85E+05	
+2,4-Pentanedione	123-54-6	1.91					3.16E-02	D	4		69	1.34E+04	4.25E+05
Mefruside	7195-27-9	1.91						D	4		71	1.34E+04	
Cyclohexane	110-82-7	1.92					2.19E+01	D	3		82	4.27E+04	1.95E+03
D	4		89	5.25E+04	2.40E+03
D	5	3: 55 42 41	86	3.56E+04	1.63E+03
E	1	2: 41 84	76	1.34E+04	6.13E+02
2,3,4-trimethylpentane	565-75-3	1.98						D	3	5: 70 57 39 55 84	74	4.45E+05	
(S)-2-propylpiperidine	458-88-8	1.99						E	1		66	3.60E+03	
2-ethyl-1-butanol	97-95-0	2.00		Sweet, Musty, Alcoholic			2.34E-01	D	4	3: 84 70 39	75	1.29E+05	5.52E+05
D	5		74	3.28E+04	1.40E+05
+Cyclohexanone	108-94-1	2.17		Minty, Acetone			7.08E-01	D	4	4: 41 55 98 72	65	9.49E+03	1.34E+04
Nimorazole	6506-37-2	2.19						D	3	1: 100	72	8.60E+03	
2-(diethylamino)-1-phenyl-1-propanone	90-84-6	2.19						D	3	1: 100	66	8.60E+03	
+Heptane	142-82-5	2.22	Alkane	Sweet, Ethereal			9.77E+00	D	3	12: 43 71 41 100 56 55 70 54 39 42 85 40	98	1.40E+06	1.43E+05
+2-methyl-3-pentanone	565-69-5	2.22	Mint	Mint				D	4	3: 100 57 41	67	1.24E+04	
1,2-diethyl hydrazine	1615-80-1	2.31						D	4	6: 88 70 89 73 87 60	73	5.77E+05	
+Ethylacetate	141-78-6	2.31	Pineapple	Ethereal, Fruity, Sweet, Weedy, Green			2.63E+00	D	1	9: 43 61 70 73 62 71 60 89 55	99	3.08E+06	1.17E+06
D	3	6: 61 70 73 62 90 60	99	4.00E+06	1.52E+06
D	4	10: 43 61 42 70 88 45 73 62 87 41	99	3.29E+06	1.25E+06
D	5	7: 70 88 73 42 74 62 59	99	2.31E+06	8.80E+05
+2-butanone	78-93-3	2.33	Ether	Ethereal, Fruity, Camphor			7.76E+00	D	3	4: 43 61 45 60	75	4.36E+06	5.61E+05
D	4	3: 72 57 39	67	2.54E+05	3.27E+04
D	5	5: 43 61 45 73 89	76	2.23E+06	2.88E+05
Methyl thiocyanate	556-64-9	2.33	Sulfur	Sulfury, Onion			1.55E-01	D	5	4: 42 73 46 60	66	3.15E+05	2.03E+06
+Ethanol	64-17-5	2.34	Sweet	Alcoholic			2.88E+01	D	1	2: 45 73	87	4.09E+05	1.42E+04
D	2		98	1.58E+05	5.47E+03
+Isopropyl alcohol	67-63-0	2.34		Alcohol, Musty, Woody			1.02E+01	D	2	1: 45	81	2.69E+05	2.63E+04
D	4		80	5.72E+05	5.59E+04
D	5		82	2.92E+05	2.86E+04
+Formic acid	64-18-6	2.34		Acetic			2.82E+01	D	1	2: 45 73	78	1.00E+06	3.56E+04
D	2		70	8.70E+04	3.09E+03
Nitrogen dioxide	10102-44-0	2.34					1.86E-01	D	1	1: 46	75	1.36E+05	7.28E+05
D	5	2: 46 47	76	6.16E+03	3.31E+04
methylhydrazine	60-34-4	2.35						D	1	1: 46	78	1.95E+05	
D	2	2: 45 46	77	9.05E+04	
Acetic acid ethenyl ester	108-05-4	2.41						D	5	1: 86	68	8.39E+04	
+Methylene chloride	75-09-2	2.41					2.82E+01	D	3	4: 84 49 48 35	97	2.87E+05	1.02E+04
D	4	6: 84 49 86 51 35 47	93	2.60E+05	9.24E+03
D	5	6: 49 44 57 84 48 35	93	1.22E+05	4.34E+03
Tolycaine	3686-58-6	2.43						D	5	3: 86 47 35	67	2.41E+04	
+2-Pentanone	107-87-9	2.43	Ether, Fruit	Sweet, Fruity, Ethereal, Wine, Banana, Woody			1.55E+00	D	5	4: 86 49 84 43	72	6.97E+04	4.50E+04
Amitrole	61-82-5	2.49						D	3	3: 84 46 57	79	2.35E+04	
Piperoxan	59-39-2	2.60						D	3	5: 98 85 84 69 82	67	3.15E+04	
Methyl cyclohexane	108-87-2	2.61						D	3	5: 83 56 41 69 39	94	1.24E+05	
D	4	5: 98 55 83 82 56	84	1.14E+05	
D	5	4: 83 69 82 55	75	2.64E+04	
+n-Propyl acetate	109-60-4	2.68	Fruit, Apple, Banana	Solvent, Celery, Fruity, Fusel, Raspberry, Pear			5.75E-01	D	1	9: 43 61 70 73 62 71 60 89 55	69	4.99E+06	8.68E+06
D	3	9: 41 33 59 60 39 72 57 74 35	97	6.43E+06	1.12E+07
D	4	10: 43 61 42 70 88 45 73 62 87 41	69	3.29E+06	5.71E+06
D	5	7: 70 88 73 42 74 62 59	69	2.31E+06	4.02E+06
+1-Heptanol	111-70-6	2.77	Chemical, Green	Musty, Leafy, Violet, Herbal, Green, Sweet, Woody, Peony			2.51E-02	D	3		73	3.82E+04	1.52E+06
Ethanedinitrile	460-19-5	3.00						D	5	2: 52 61	74	1.75E+03	
Benzene	71-43-2	3.02		Aromatic			3.63E+00	D	1		93	1.32E+05	3.64E+04
D	3	4: 78 50 77 79	74	2.71E+04	7.47E+03
2,5-dimethyl hexane	592-13-2	3.17						D	3	6: 70 53 43 39 99 56	84	9.76E+05	
3-methylheptane	589-81-1	3.35						D	3		93	1.29E+05	
Sorbic Acid	110-44-1	3.56						D	3		67	6.14E+04	
+Isothiocyanato methane	556-61-6	3.76		Pungent, Mustard, Horseradish				D	3	4: 73 40 72 63	67	9.72E+05	
Chloroform	67-66-3	3.77						D	4	4: 83 48 61 85	79	2.12E+05	
D	5		79	7.16E+04	
Ethylenediamine	107-15-3	3.93						D	1		68	9.65E+04	
+1,1-dimethyl-hydrazine	57-14-7	3.95						D	1	3: 59 42 60	74	2.19E+04	
D	2		79	4.75E+04	
3-pentanol	584-02-1	3.95	Fruit	Herbal			4.68E-01	D	2		66	4.75E+04	1.02E+05
Hydrazine	302-01-2	3.96					3.00E+00	D	1		79	1.44E+04	4.79E+03
D	2	1: 33	77	1.47E+03	4.89E+02
D	3	1: 33	78	8.97E+04	2.99E+04
D	4	2: 111 33	78	6.05E+03	2.02E+03
+Octane	111-65-9	4.00	Alkane	Gasoline			5.75E+00	D	3		91	1.90E+05	3.30E+04
Tetrahydrofurfuryl acetate	637-64-9	4.07		Sweet, Fruity, Brown, Rum, Ether, Caramel				D	3	2: 71 39	77	1.98E+05	
Isobutyl acetate	110-19-0	4.86	Fruit, Apple, Banana	Sweet, Fruity, Ethereal, Banana, Tropical			4.79E-01	D	1		91	2.03E+05	4.24E+05
+Isobutyric acid	79-31-2	4.88	Rancid, Butter, Cheese				1.95E-02	D	1	2: 43 41	72	2.84E+05	1.46E+07
+Toluene	108-88-3	5.05	Paint	Sweet			1.55E+00	D	3	16: 91 65 93 89 39 50 38 62 77 43 45 74 90 61 46 88	100	5.76E+06	3.72E+06
+Phenylethyl alcohol	60-12-8	5.05	Honey, Spice, Rose, Lilac	Floral			1.70E-02	D	3	16: 91 65 93 89 39 50 38 62 77 43 45 74 90 61 46 88	75	5.76E+06	3.39E+08
+1-butanol	71-36-3	6.15	Medicine, Fruit	Fermented			4.90E-01	D	1		80	7.81E+03	1.59E+04
D	2	4: 43 42 56 41	68	7.76E+04	1.58E+05
D	5		73	1.90E+04	3.88E+04
+Isobutanol	78-83-1	6.17	Wine, Solvent, Bitter	Ethereal, Winey				D	1	18: 43 41 42 33 39 74 40 72 56 57 38 59 44 53 73 60 51 37	97	1.29E+07	
D	3	3: 42 41 43	73	2.60E+05	
D	4	6: 43 57 41 42 56 39	68	1.23E+05	
D	5	3: 39 42 41	65	5.14E+04	
Propanoic acid, anhydride	123-62-6	6.49						D	2	1: 57	69	2.88E+04	
D	3	1: 57	76	8.26E+04	
D	5	1: 57	65	3.15E+04	
4-methyl-3-penten-2-one	141-79-7	6.65	Sweet, Chemical	Pungent, Earthy, Vegetable, Acrylic			5.62E-02	D	3	5: 83 56 41 69 39	79	1.24E+05	2.21E+06
D	4	5: 98 55 83 82 56	84	6.12E+04	1.09E+06
D	5	3: 98 83 55	82	2.31E+04	4.11E+05
+Decane	124-18-5	6.66	Alkane				7.41E-01	D	3		69	6.62E+04	8.93E+04
E	1		82	3.45E+04	4.66E+04
+Isoamyl alcohol	123-51-3	7.52	Whiskey, Malt, Burnt	Fusel oil, Alcoholic, Whiskey, Fruity, Banana			4.47E-02	D	1		69	2.43E+04	5.45E+05
D	4	3: 55 70 53	66	1.40E+04	3.14E+05
Amyl alcohol	71-41-0	7.54	Balsamic	Fusel, Oil, Sweet, Balsam			4.68E-01	D	1		77	2.43E+04	5.20E+04
D	4	3: 55 70 53	71	1.40E+04	3.00E+04
D	5	2: 42 55	72	2.96E+04	6.34E+04
+p-xylene	106-42-3	7.65					4.90E-01	D	3	2: 91 105	83	8.07E+04	1.65E+05
α-pinene	80-56-8	7.90	Pine, Turpentine	Herbal			6.92E-01	D	1		75	1.18E+04	1.70E+04
α-phellandrene	99-83-2	7.91	Turpentine, Mint, Spice	Terpenic				D	1		79	1.18E+04	
+Camphene	79-92-5	10.21	Camphor	Woody				D	1		67	1.18E+04	
p-ethyltoluene	622-96-8	10.25						D	3	5: 120 105 91 155 136	76	4.89E+04	
2-ethyltoluene	611-14-3	10.61						D	3	4: 105 154 77 91	75	5.10E+04	
2,2,5-trimethylhexane	3522-94-9	10.67						D	3	7: 57 70 112 83 69 72 155	84	8.52E+05	
+1-hexanol	111-27-3	10.73	Resin, Flower, Green	Herbal			4.37E-02	D	5	4: 69 56 41 42	66	7.45E+04	1.71E+06
Diacetone alcohol	123-42-2	10.79					8.91E-01	D	1		89	1.90E+05	2.14E+05
D	4	10: 43 59 58 42 41 57 98 38 45 61	93	2.46E+06	2.76E+06
D	5	7: 43 59 58 39 55 207 53	92	1.22E+06	1.37E+06
1,3,5-trimethylbenzene	108-67-8	11.02						D	3	7: 105 119 120 106 43 77 102	83	1.34E+05	
+Piperidine	110-89-4	11.20		Animal			3.72E-01	D	3		79	1.29E+05	3.46E+05
2,4,5-trimethylbenzenamine	137-17-7	11.30						D	3	1: 120	73	1.13E+05	
+Durene	95-93-2	11.36	Rancid, Sweet	Rancid			2.63E-02	D	1	2: 134 119	67	3.08E+04	1.17E+06
Isodurene	527-53-7	11.37						D	1	2: 134 119	68	1.76E+04	
D	3	2: 119 134	70	4.14E+04	
1-ethyl-2,4-dimethylbenzene	874-41-9	11.38						D	3	2: 119 134	70	8.63E+04	
1,2,3,4-tetramethylbenzene	488-23-3	11.38		Gasoline, Sweet			2.63E-02	D	1		78	2.05E+04	7.81E+05
								D	3	2: 119 134	65	8.63E+04	3.28E+06
+p-cymene	99-87-6	11.38	Solvent, Gasoline, Citrus	Terpenic			2.14E-03	D	1		81	2.05E+04	9.61E+06
								D	3	2: 119 134	67	8.63E+04	4.04E+07
N,N-dimethylbenzenamine	121-69-7	11.41						D	3	9: 120 105 121 103 79 91 97 77 122	82	2.62E+05	
3-phenyl propyl isobutyrate	103-58-2	11.41		Sweet, Fruity, Balsam				D	3	3: 118 117 141	69	1.09E+04	
3-phenyl propyl acetate	122-72-5	11.41		Sweet, Balsam, Storax, Spicy, Cinnamon				D	3	3: 118 117 141	67	1.09E+04	
2,4,6-trimethylbenzenamine	88-05-1	11.42						D	1		84	8.19E+04	
D	3	1: 120	71	1.13E+05	
p-aminotoluene	106-49-0	11.53						D	3	4: 107 43 106 93	66	4.93E+04	
3,5-dimethylbenzenamine	108-69-0	12.00						D	3		78	1.88E+05	
2,4,6-trimethylpyridine	108-75-8	12.00						D	3	7: 120 121 77 56 66 57 122	73	3.46E+05	
+Acetic acid	64-19-7	12.10	Sour	Acidic			1.45E-01	D	3	5: 43 60 42 41 61	99	4.73E+07	3.27E+08
D	4		100	7.03E+07	4.87E+08
D	5	6: 45 43 40 62 56 47	100	5.47E+07	3.78E+08
+o-xylene	95-47-6	13.02	Geranium	Geranium			8.51E-01	D	3		73	6.49E+03	7.63E+03
Benzo[b]thiophene	95-15-8	13.39		Solvent, Rubbery, Earthy				D	1	1: 134	65	5.14E+04	
p-Hydroxyamphetamine acetate	96750-10-6	13.39						D	1		66	5.86E+04	
+Nonanal	124-19-6	13.64	Fat, Citrus, Green	Aldehydic		1.00E-03	2.24E-03	D	1		69	1.28E+04	5.73E+06
E	1		67	9.94E+03	4.44E+06
+2-ethylhexanol	104-76-7	13.81	Rose, Green	Citrus			2.45E-01	D	1		88	2.79E+05	1.14E+06
D	3	14: 42 98 70 112 39 58 113 84 69 72 54 68 99 51	98	4.65E+06	1.90E+07
+Methyl vinyl ketone	78-94-4	13.90		Sweet				D	1	3: 41 70 55	69	6.55E+04	
+Propanoic acid	79-09-4	13.90	Pungent, Rancid, Soy	Pungent, Acidic, Cheesy, Vinegar			3.55E-02	D	1	9: 74 44 55 38 56 57 46 37 58	67	1.92E+06	5.40E+07
D	4	2: 73 74	94	2.04E+05	5.76E+06
D	5		96	8.40E+04	2.37E+06
Propylene glycol	57-55-6	13.99						D	1	2: 45 73	66	4.09E+05	
D	2		74	1.58E+05	
D	4		96	5.72E+05	
D	5	2: 45 55	93	2.58E+05	
+Benzaldehyde	100-52-7	14.08	Almond, Burnt sugar	Fruity		3.00E-03	4.17E-02	D	1		97	7.61E+05	1.83E+07
D	2	4: 77 105 106 51	88	3.14E+04	7.52E+05
D	3	10: 107 76 74 52 39 49 108 73 37 64	98	4.88E+06	1.17E+08
D	4	6: 105 77 106 51 50 52	95	2.19E+05	5.25E+06
D	5		90	3.95E+04	9.47E+05
Indane	496-11-7	14.10						D	3	3: 118 117 141	67	1.09E+04	
Isobutyrophenone	611-70-1	14.10		Green				D	1	13: 105 77 51 78 106 74 75 49 38 50 76 52 39	76	1.35E+06	
**E**	1	7: 37 105 119 121 118 93 62	80	4.77E+06	
+Nonane	111-84-2	14.13	Alkane	Gasoline			1.26E+00	D	3		86	5.80E+04	4.61E+04
2-chloroacetophenone	532-27-4	14.14		Apple blossom			2.57E-02	D	1	13: 105 77 51 78 106 74 75 49 38 50 76 52 39	87	1.35E+06	5.26E+07
D	2		76	4.04E+04	1.57E+06
D	3	10: 107 76 74 52 39 49 108 73 37 64	76	4.88E+06	1.90E+08
D	4	6: 105 77 106 51 50 52	74	2.19E+05	8.52E+06
D	5		69	3.95E+04	1.54E+06
E	1	7: 37 105 119 121 118 93 62	92	3.56E+05	1.38E+07
+Undecane	1120-21-4	14.14	Alkane				1.17E+00	D	3	5: 127 53 55 39 72	78	9.14E+05	7.78E+05
2,2-dimethylbutane	75-83-2	14.15						D	3	3: 41 71 56	84	2.69E+05	
+Dodecane	112-40-3	14.15	Alkane	Alkane			2.04E+00	E	1		95	2.59E+05	1.27E+05
+Tridecane	629-50-5	14.17	Alkane	Alkane			2.14E+00	D	1	8: 41 56 57 86 85 99 112 70	74	3.03E+05	1.42E+05
D	3	8: 85 127 57 55 82 70 128 126	76	6.23E+05	2.92E+05
Octyl acetate	112-14-1	14.20		Green, Earthy, Mushroom, Herbal, Waxy			3.98E-03	D	1	4: 56 57 55 43	76	7.44E+04	1.87E+07
N-Nitrosodimethylamine	62-75-9	14.66						D	5	3: 74 43 57	70	2.72E+05	
+Ethyl lactate	97-64-3	14.90	Fruit	Sharp, Tart, Fruity, Buttery, Butterscotch			1.62E+00	D	5	2: 45 55	68	4.23E+05	2.61E+05
2-Hydroxyethylhydrazine	109-84-2	14.91						D	4		65	5.72E+05	
D	5		67	5.15E+04	
+Ethyl octanoate	106-32-1	15.23	Fruit, Fat	Fruity, Wine, Waxy, Sweet, Apricot, Banana, Brandy, Pear			5.75E-04	E	1	9: 101 43 73 102 88 61 60 129 168	87	9.77E+04	9.77E+04
tetrahydro-2-methyl-2-furanol	7326-46-7	15.59						D	3	2: 71 69	77	6.97E+04	
	D	4		76	1.27E+04	
+1-methyl-1H-pyrrole	96-54-8	15.72		Smoky, Woody, Herbal				D	2	1: 81	69	2.94E+04	
2-ethoxyethanol	110-80-5	15.79					1.23E+00	D	1	3: 59 60 37	74	1.19E+05	9.65E+04
Hexestrol	84-16-2	15.85						D	1		73	8.19E+04	
Methyl benzoate	93-58-3	16.30	Prune, Lettuce, Herb, Sweet	Phenolic			1.07E-01	D	1	11: 105 77 136 76 137 106 39 49 75 74 91	99	1.81E+06	1.69E+07
E	1	10: 105 77 92 49 52 152 64 181 127 141	100	1.35E+08	1.26E+09
Cumene	98-82-8	16.49					2.40E-02	D	3	5: 105 135 120 77 78	77	2.84E+05	1.18E+07
+Acetophenone	98-86-2	16.49	Musty, Flower, Almond	Floral		6.50E-02	3.63E-01	D	3	5: 105 135 120 77 78	93	2.84E+05	7.81E+05
3-ethyltoluene	620-14-4	16.50						D	3	4: 78 105 120 106	78	1.52E+05	
2,2,4-trimethylpentane	540-84-1	16.53						D	1	4: 56 57 55 43	66	8.44E+04	
D	3	3: 41 57 56	88	2.57E+05	
2-ethyl-5-methylpyrazine	13360-64-0	16.81	Fruit, Sweet	Coffee bean, Nutty				D	3	3: 121 122 81	73	3.39E+04	
γ-hexalactone	695-06-7	17.20	Coumarin, Sweet	Tonka				D	3	4: 56 85 69 51	68	2.89E+05	
2-ethyl-3,5-dimethylpyridine	1123-96-2	17.90						D	1		91	5.86E+04	
D	3	4: 107 135 134 70	86	1.49E+05	
+α-α-Dimethylbenzenemethanol	617-94-7	18.05		Mild, Green, Sweet, Earthy				D	3	6: 122 105 78 77 136 102	89	1.71E+05	
p-methoxyphenylacetone	122-84-9	18.07		Sweet, Fruity, Spicy, Anisic, Balsam				D	3		68	9.60E+04	
3-methylhexane	589-34-4	18.33						D	1		70	4.60E+03	
D	3	7: 43 70 41 56 39 42 100	97	5.33E+05	
+Tetradecane	629-59-4	18.34	Alkane	Mild, Waxy				D	1	5: 198 140 154 82 100	98	3.83E+06	
1-undecanol	112-42-5	18.37	Mandarin	Waxy			6.76E-02	E	1	4: 111 83 97 106	74	2.87E+04	4.24E+05
Nitrocyclohexane	1122-60-7	19.50						D	3	5: 83 56 41 69 39	74	2.15E+05	
β-caryophyllene	87-44-5	19.68	Wood, Spice	Spice		6.40E-02		D	1		66	1.51E+04	2.36E+05
D	2		69	1.18E+04	1.84E+05
D	3		85	7.37E+04	1.15E+06
+Pentadecane	629-62-9	20.28	Alkane	Waxy				D	1	8: 41 56 57 86 85 99 112 70	86	1.74E+05	
+Butanoic acid, butyl ester	109-21-7	20.97		Fruity, Banana, Pineapple, Sweet				D	1		76	3.03E+04	
Longifolene	475-20-7	21.28		Wood				D	3		74	7.37E+04	
Toluene-2,4-diamine	95-80-7	23.91						D	3	3: 121 122 81	72	3.39E+04	
2,3,6-trimethylpyridine	1462-84-6	23.96						D	3		74	1.06E+05	

If two references for ODTs are available, ODT from Devos, et al. [5] is used to calculate OAV. RT = Retention Time. ODT = Odor Detection Threshold. Code, see Section 1.5. Models = significant ions used for identification/semi-quantitation, # before colon is number of significant ions, #’s after colon are *m/z*. Net % match as calculated using AMDIS and target specialty mass spectral libraries. PAC = Peak Area Counts, and refers to relative abundance as given by the mass spectral detector. OAV = Odor Activity Value, and is calculated as ratio of PAC: OAV. Underlined items highlight the compounds found in Pseudo Scent Cocaine. + Compounds indicate confirmation with reference standards, matching retention time and spectra.

**Table 5:** Summary of VOCs emitted from all illicit heroin samples (sample code F in Section 1.5) and Sigma Pseudo™ Narcotic Scent Heroin formulation (sample code G in Section 1.5) and sampled over 1 h at room temperature. **Sigma Pseudo™ Narcotic Scent Heroin formulation is indicated by underlined fonts.**

**Compound**	**CAS**	**RT (min)**	**Published Descriptors**		**Published ODT (ppm)**		**Sample code**		**Models**	**Net % match**	**PAC**	**OAV**
			**Flavornet**[7]	**TGSC**[8]	**LRI & Odour**[6]	**Devos et al**. [5]						
Ethylene oxide	75-21-8	1.06				8.51E+02	F	1	3: 44 45 46	66	3.54E+06	4.16E+03
G	1	4: 44 45 46 43	68	1.75E+06	2.06E+03
+2-nitropropane	79-46-9	1.12				7.24E+00	F	2	3: 43 41 58	74	1.30E+04	1.80E+03
Methyl chloride	74-87-3	1.15					F	1	2: 50 52	73	1.00E+04	
+Isobutanol	78-83-1	1.19	Wine, Solvent, Bitter	Ethereal, Winey			F	1		70	4.81E+04	
F	2	6: 42 57 43 41 56 39	68	8.76E+04	
Hexane	110-54-3	1.19	Alkane			2.19E+01	F	1	6: 43 41 57 42 56 39	82	7.74E+04	3.54E+03
F	2	6: 42 57 43 41 56 39	82	8.76E+04	4.00E+03
Isobutane	75-28-5	1.23				1.00E+01	F	1	10: 43 42 41 57 72 39 55 56 38 58	83	8.96E+05	8.96E+04
F	2	13: 43 42 41 72 39 57 56 63 53 38 73 58 37	83	1.18E+06	1.18E+05
Isobutyraldehyde	78-84-2	1.23	Pungent, Malt, Green	Spicy		4.07E-02	F	1	10: 43 42 41 57 72 39 55 56 38 58	78	8.96E+05	2.20E+07
F	2	13: 43 42 41 72 39 57 56 63 53 38 73 58 37	77	1.18E+06	2.89E+07
4-methyldecane	2847-72-5	1.39					F	1	4: 71 57 41 72	67	4.33E+05	
F	2	9: 43 42 41 39 55 85 53 38 69	66	4.19E+05	
2-methylpentane	107-83-5	1.39					F	1	4: 71 57 41 72	97	4.33E+05	
F	2	9: 43 42 41 39 55 85 53 38 69	97	4.19E+05	
Ethylenimine	151-56-4	1.40					F	2	9: 43 42 41 39 55 85 53 38 69	86	3.48E+05	
2,3-dimethylbutane	79-29-8	1.40					F	2	9: 43 42 41 39 55 85 53 38 69	81	3.48E+05	
3,4,5-trimethyl-1-hexene	56728-10-0	1.40					F	1		68	2.72E+05	
F	2	8: 71 43 57 56 70 51 39 86	67	3.94E+05	
3-methylhexane	589-34-4	1.40					F	2	8: 71 43 57 56 70 51 39 86	75	2.78E+05	
+1-butanol	71-36-3	1.42	Medicine, Fruit	Fermented		4.90E-01	F	1	6: 43 41 57 42 56 39	66	7.74E+04	1.58E+05
F	2		81	1.12E+04	2.29E+04
G	1	4: 39 41 69 43	72	6.39E+04	1.30E+05
3-methylpentane	96-14-0	1.45					F	1	3: 71 56 57	87	5.52E+04	
F	2		88	2.45E+04	
2-methylaziridine	75-55-8	1.49					F	1	3: 71 56 57	79	5.52E+04	
F	2	2: 41 56	80	3.37E+04	
Isocyanatomethane	624-83-9	1.52					F	1		77	1.32E+04	
Tolycaine	3686-58-6	1.52					F	1	1: 86	67	8.96E+02	
+Propene	115-07-1	1.65				5.25E+01	F	2	3: 42 39 41	73	3.41E+04	6.50E+02
+Butane	106-97-8	1.66				2.04E+02	F	1	4: 58 43 42 38	74	1.45E+05	7.10E+02
F	2		79	2.36E+05	1.15E+03
+Acetone	67-64-1	1.66		Solvent		1.45E+01	F	1	4: 58 43 42 38	92	1.45E+05	1.00E+04
F	2	5: 43 58 39 37 38	97	3.14E+05	2.18E+04
Hydrazine	302-01-2	1.97				3.00E+00	F	1	1: 33	78	1.19E+03	3.97E+02
Cyclohexane	110-82-7	1.98				2.19E+01	G	1	9: 39 84 56 42 55 69 85 50 54	96	2.71E+05	1.24E+04
+Ethylacetate	141-78-6	2.32	Pineapple	Ethereal, Fruity, Sweet, Weedy, Green		2.63E+00	F	2		96	2.41E+05	9.17E+04
Propylene glycol	57-55-6	2.33					F	1	2: 43 45	69	5.16E+04	
F	2	3: 45 61 44	65	8.74E+04	
+Isopropyl alcohol	67-63-0	2.33		Alcohol, Musty, Woody		1.02E+01	F	1	2: 43 45	69	2.14E+04	2.09E+03
+Ethanol	64-17-5	2.33	Sweet	Alcoholic		2.88E+01	F	2	3: 45 61 44	68	6.90E+04	2.39E+03
+Acetic anhydride	108-24-7	3.66		Sharp, Vinegar		5.89E-01	F	1	2: 43 42	76	6.24E+03	1.06E+04
F	2	4: 43 37 42 38	71	1.43E+05	2.43E+05
G	1	1: 43	69	3.64E+04	6.18E+04
nitrocyclohexane	1122-60-7	10.29					G	1	3: 83 55 41	74	2.39E+04	
m-cymene	535-77-3	11.33					G	1		93	4.08E+04	
1-(3-methylphenyl)-ethanone	585-74-0	11.34					G	1		86	2.84E+04	
tert-butyl-benzene	98-06-6	11.34					G	1		88	2.84E+04	
1,2,3,4-tetramethylbenzene	488-23-3	11.35		Gasoline, Sweet		2.63E-02	F	1	3: 120 119 134	66	2.94E+04	1.12E+06
							G	1		86	4.08E+04	1.55E+06
+p-cymene	99-87-6	11.35	Solvent, Gasoline, Citrus	Terpenic		2.14E-03	F	1	3: 120 119 134	65	2.94E+04	1.37E+07
G	1		91	4.08E+04	1.91E+07
Isodurene	527-53-7	11.37					F	1	3: 120 119 134	69	2.94E+04	
+Acetic acid	64-19-7	12.09	Sour	Acidic		1.45E-01	F	1	5: 43 60 41 59 47	97	5.74E+07	3.97E+08
F	2		99	2.62E+05	1.81E+06
G	1	5: 45 43 60 46 105	100	5.84E+07	4.04E+08
Nitrogen dioxide	10102-44-0	12.29				1.86E-01	G	1	1: 46	76	9.21E+02	4.95E+03
+Furfural	98-01-1	12.71	Bread, Almond, Sweet	Sweet, Woody, Almond, Baked bread		7.76E-01	F	2		93	3.22E+04	4.15E+04
Fenbendazole	43210-67-9	12.98					F	1	3: 267 269 268	66	5.95E+04	
+Propanoic acid	79-09-4	13.91	Pungent, Rancid, Soy	Pungent, Acidic, Cheesy, Vinegar		3.55E-02	F	1		94	9.03E+04	2.54E+06
Propanoic acid, anhydride	123-62-6	13.91					F	2	5: 57 209 193 82 69	68	3.17E+03	
G	1	1: 57	66	4.74E+03	
Benzaldehyde	100-52-7	14.10	Almond, Burnt sugar	Fruity	3.00E-03	4.17E-02	G	1	2: 105 77	76	5.40E+04	1.30E+06
2-chloroacetophenone	532-27-4	14.10		Apple blossom		2.57E-02	G	1	2: 105 77	77	5.40E+04	2.10E+06
Isobutyrophenone	611-70-1	14.10		Green			G	1	2: 105 77	66	3.06E+04	
Ethyl cyclohexane	1678-91-7	15.20					G	1	1: 83	70	7.33E+04	
Butyric acid	107-92-6	15.53	Rancid, Cheese, Sweat	Sharp, Acetic, Cheese, Butter, Fruit		3.89E-03	F	1	3: 60 42 37	95	4.20E+05	1.08E+08
+Pentanoic acid	109-52-4	15.53	Sweat	Sickening, Putrid, Acidic, Sweaty, Rancid		4.79E-03	F	1	5: 60 45 73 43 39	89	3.45E+05	7.22E+07
2,2-dimethylbutane	75-83-2	15.87					F	1		82	1.06E+04	
Methyl benzoate	93-58-3	16.26	Prune, Lettuce, Herb, Sweet	Phenolic		1.07E-01	G	1		97	1.74E+05	1.63E+06
+Toluene	108-88-3	19.16	Paint	Sweet		1.55E+00	F	2		80	2.84E+04	1.84E+04
+Dimethylsulfone	67-71-0	20.11	Sulfur, Burnt	Sulfurous, Burnt			F	2	2: 79 62	96	1.94E+05	
Methyl formate	107-31-3	22.87		Fruity, Plum		9.33E+01	G	1	1: 60	73	1.83E+03	1.96E+01
Diethyl Phthalate	84-66-2	27.46					F	1		69	9.71E+03	

If two references for ODTs are available, ODT from Devos, et al. [5] is used to calculate OAV. RT=Retention Time. ODT=Odor Detection Threshold. Code, see Section 1.5. Models=significant ions used for identification/semi-quantitation, # before colon is number of significant ions, #’s after colon are *m/z*. Net % match as calculated using AMDIS and target specialty mass spectral libraries. PAC = Peak Area Counts, and refers to relative abundance as given by the mass spectral detector. OAV = Odor Activity Value, and is calculated as ratio of PAC: OAV. Underlined items highlight the compounds found in Pseudo Scent Heroin. + Compounds indicate confirmation with reference standards, matching retention time and spectra.

**Table 6:** Comparing rank of top 10 most concentrated VOCs with the calculated OAV in all marijuana samples. Bolded font signifies 1 g real marijuana (sample code A6/A7). Underlined font signifies 1 g surrogate marijuana (sample code C1–C3).

**Compound**	**CAS**	**Sample code**		**Rank conc.**	**Rank OAV**	**Change in ranking (Rank Conc. – Rank OAV)**
Limonene	138-86-3	A	1	1	5	−4
Limonene	138-86-3	A	2	1	6	−5
Isobutane	75-28-5	A	3	1	6	−5
Ethylene oxide	75-21-8	A	4	1	17	−16
(+)-sativene	3650-28-0	A	5	1	No ODT	
**Acetic acid**	**64-19-7**	**A**	**6**	**1**	**2**	−**1**
**Benzyl alcohol**	**100-51-6**	**A**	**7**	**1**	**No ODT**	
Tyramine	51-67-2	B	1	1	No ODT	
β-pinene	18172-67-3	B	2	1	No ODT	
Acetic acid	64-19-7	B	3	1	2	−1
Butyl formate	592-84-7	B	4	1	No ODT	
γ-terpinene	99-85-4	C	1	1	No ODT	
γ-terpinene	99-85-4	C	2	1	No ODT	
Camphene	79-92-5	C	3	1	No ODT	
Camphene	79-92-5	A	1	2	No ODT	
Camphene	79-92-5	A	2	2	No ODT	
2-methylpentane	107-83-5	A	3	2	No ODT	
Acetone	67-64-1	A	4	2	12	−10
Benzyl alcohol	100-51-6	A	5	2	No ODT	
**Methylisohexenyl ketone**	**110-93-0**	**A**	**6**	**2**	**3**	−**1**
**Acetone**	**67-64-1**	**A**	**7**	**2**	**21**	−**19**
β-pinene	18172-67-3	B	1	2	No ODT	
Myrcene	123-35-3	B	2	2	1	+1
Methylisohexenyl ketone	110-93-0	B	3	2	3	−1
Acetone	67-64-1	B	4	2	14	−12
Ethyl benzene	100-41-4	C	1	2	3	−1
Terpinolene	586-62-9	C	2	2	3	−1
γ-terpinene	99-85-4	C	3	2	No ODT	
Isobornyl thiocyanoacetate	115-31-1	A	1	3	No ODT	
Isobornyl thiocyanoacetate	115-31-1	A	2	3	No ODT	
3,4,5-trimethyl-1-hexene	56728-10-0	A	3	3	No ODT	
Limonene	138-86-3	A	4	3	4	−1
Tyramine	51-67-2	A	5	3	No ODT	
**β-caryophyllene**	**87-44-5**	**A**	**6**	**3**	**4**	−**1**
**Tyramine**	**51-67-2**	**A**	**7**	**3**	**No ODT**	
Myrcene	123-35-3	B	1	3	1	+2
Limonene	138-86-3	B	2	3	4	−1
β-caryophyllene	87-44-5	B	3	3	4	−1
3-pentanol	584-02-1	B	4	3	5	−2
m-cymene	535-77-3	C	1	3	No ODT	
Ethyl benzene	100-41-4	C	2	3	5	−2
Terpinolene	586-62-9	C	3	3	3	0
β-pinene	18172-67-3	A	1	4	No ODT	
Isobutane	75-28-5	A	2	4	13	−9
4-methyldecane	2847-72-5	A	3	4	No ODT	
Camphene	79-92-5	A	4	4	No ODT	
Isobutane	75-28-5	A	5	4	9	−5
**Longifolene**	**475-20-7**	**A**	**6**	**4**	**No ODT**	
**Isobutane**	**75-28-5**	**A**	**7**	**4**	**22**	−**18**
Ethylene oxide	75-21-8	B	1	4	28	−24
Camphene	79-92-5	B	2	4	No ODT	
Longifolene	475-20-7	B	3	4	No ODT	
tert-butanol	75-65-0	B	4	4	No ODT	
p-cymene	99-87-6	C	1	4	1	+3
m-cymene	535-77-3	C	2	4	No ODT	
m-cymene	535-77-3	C	3	4	No ODT	
Isobutane	75-28-5	A	1	5	11	−6
Isobutyraldehyde	78-84-2	A	2	5	3	+2
Ethylene oxide	75-21-8	A	3	5	19	−14
2,2,4-trimethylpentane	540-84-1	A	4	5	No ODT	
γ-gurjunene	22567-17-5	A	5	5	No ODT	
**Acetone**	**67-64-1**	**A**	**6**	**5**	**22**	−**17**
**Butane**	**106-97-8**	**A**	**7**	**5**	**34**	**−29**
Benzyl alcohol	100-51-6	B	1	5	No ODT	
α-phellandrene	99-83-2	B	2	5	No ODT	
Fenchyl alcohol	1632-73-1	B	3	5	No ODT	
Propylamine	107-10-8	B	4	5	1	+4
1,2,3,4-tetramethylbenzene	488-23-3	C	1	5	2	+3
p-cymene	99-87-6	C	2	5	1	+4
p-cymene	99-87-6	C	3	5	1	+4
Isobutyraldehyde	78-84-2	A	1	6	1	+5
β-pinene	18172-67-3	A	2	6	No ODT	
Limonene	138-86-3	A	3	6	4	+2
Methylene chloride	75-09-2	A	4	6	15	−9
α-humulene	6753-98-6	A	5	6	17	−11
**Butane**	**106-97-8**	**A**	**6**	**6**	**31**	**−25**
**α-phellandrene**	**99-83-2**	**A**	**7**	**6**	**No ODT**	
Butane	106-97-8	B	1	6	25	−19
α-pinene	80-56-8	B	2	6	5	+1
Butane	106-97-8	B	3	6	35	−29
Ethylene oxide	75-21-8	B	4	6	23	−17
1-ethyl-3,5-dimethylbenzene	934-74-7	C	1	6	No ODT	
1,2,3,4-tetramethylbenzene	488-23-3	C	2	6	2	+4
1,2,3,4-tetramethylbenzene	488-23-3	C	3	6	2	+4
Betahistine	5638-76-6	A	1	7	No ODT	
α-pinene	80-56-8	A	2	7	10	−3
Camphene	79-92-5	A	3	7	No ODT	
Methylisohexenyl ketone	110-93-0	A	4	7	2	+5
Valencene	4630-07-3	A	5	7	No ODT	
**Fenchyl alcohol**	**1632-73-1**	**A**	**6**	**7**	**No ODT**	
**δ-3-carene**	**13466-78-9**	**A**	**7**	**7**	**20**	−**13**
Acetone	67-64-1	B	1	7	17	−10
Benzyl alcohol	100-51-6	B	2	7	No ODT	
Acetone	67-64-1	B	3	7	31	-24
Diacetone alcohol	123-42-2	B	4	7	10	−3
Isodurene	527-53-7	C	1	7	No ODT	
2-acetyl-6-methyl pyrazine	22047-26-3	C	2	7	No ODT	
2-Butanol	78-92-2	C	3	7	4	+3
α-phellandrene	99-83-2	A	1	8	No ODT	
Terpinolene	586-62-9	A	2	8	8	0
Isobutyraldehyde	78-84-2	A	3	8	2	+6
2-butanone	78-93-3	A	4	8	13	−5
3,4,5-trimethyl-1-hexene	56728-10-0	A	5	8	No ODT	
**α-humulene**	**6753-98-6**	**A**	**6**	**8**	**8**	**0**
**α-humulene**	**6753-98-6**	**A**	**7**	**8**	**8**	**0**
Isobutane	75-28-5	B	1	8	15	−7
Isobutane	75-28-5	B	2	8	12	−4
α-humulene	6753-98-6	B	3	8	9	−1
1-butanol	71-36-3	B	4	8	8	0
Dihydromethylcyclopentapyrazine	23747-48-0	C	1	8	No ODT	
Isopropyl alcohol	67-63-0	C	2	8	15	−7
Ethyl lactate	97-64-3	C	3	8	6	+2
Sabinene	3387-41-5	A	1	9	No ODT	
α-terpinene	99-86-5	A	2	9	No ODT	
Myrcene	123-35-3	A	3	9	1	+8
Methyl acetate	79-20-9	A	4	9	No ODT	
4-methyldecane	2847-72-5	A	5	9	No ODT	
**Methyl acetate**	**79-20-9**	**A**	**6**	**9**	**No ODT**	
**β-caryophyllene**	**87-44-5**	**A**	**7**	**9**	**5**	**+4**
Limonene	138-86-3	B	1	9	6	+3
Isobutyraldehyde	78-84-2	B	2	9	3	+6
Alloaromadendrene	25246-27-9	B	3	9	No ODT	
β-caryophyllene	87-44-5	B	4	9	2	+7
Ethyl lactate	97-64-3	C	1	9	8	+1
Ethyl lactate	97-64-3	C	2	9	10	−1
δ-3-carene	13466-78-9	C	3	9	12	−3
β-caryophyllene	87-44-5	A	1	10	4	+6
(+)-4-Carene	29050-33-7	A	2	10	16	−6
α-terpinyl acetate	80-26-2	A	3	10	No ODT	
Fenchyl alcohol	1632-73-1	A	4	10	No ODT	
Benzaldehyde	100-52-7	A	5	10	14	−4
**Valencene**	**4630-07-3**	**A**	**6**	**10**	**No ODT**	
**2-chloroacetophenone**	**532-27-4**	**A**	**7**	**10**	**3**	**+7**
Camphene	79-92-5	B	1	10	No ODT	
Tyramine	51-67-2	B	2	10	No ODT	
Isobornyl thiocyanoacetate	115-31-1	B	3	10	No ODT	
Longifolene	475-20-7	B	4	10	No ODT	
δ-3-carene	13466-78-9	C	1	10	14	−4
Propylene glycol	57-55-6	C	2	10	No ODT	
α-terpinene	99-86-5	C	3	10	No ODT	

Sample code, see Section 1.5, corresponding to sample identification.

ODT=odor detection threshold from Devos et al. [5].

**Table 7:** Comparing rank of top 10 most concentrated VOCs with the calculated OAV in all cocaine samples. Bolded font signifies 1 g real cocaine (sample code D4/D5). Underlined font signifies 1 g surrogate cocaine (sample code E1).

**Compound**	**CAS**	**Sample Code**		**Rank conc.**	**Rank OAV**	**Change in ranking (Rank Conc. – Rank OAV)**
Isobutanol	78-83-1	D	1	1	37	−36
Isobutyraldehyde	78-84-2	D	2	1	1	0
Acetic acid	64-19-7	D	3	1	2	−1
**Acetic acid**	**64-19-7**	**D**	**4**	**1**	**1**	**0**
**Acetic acid**	**64-19-7**	**D**	**5**	**1**	**1**	**0**
Methyl benzoate	93-58-3	E	1	1	1	0
n-Propyl acetate	109-60-4	D	1	2	8	−6
Isobutane	75-28-5	D	2	2	7	−5
n-Propyl acetate	109-60-4	D	3	2	9	−7
**Ethylene oxide**	**75-21-8**	**D**	**4**	**2**	**23**	**−21**
**n-Propyl acetate**	**109-60-4**	**D**	**5**	**2**	**2**	**0**
Isobutyrophenone	611-70-1	E	1	2	12	−10
Acetone	67-64-1	D	1	3	20	−17
4-methyldecane	2847-72-5	D	2	3	17	−14
Phenylethyl alcohol	60-12-8	D	3	3	1	+2
**n-Propyl acetate**	**109-60-4**	**D**	**4**	**3**	**4**	**−1**
**Ethylacetate**	**141-78-6**	**D**	**5**	**3**	**10**	**−7**
Ethylene oxide	75-21-8	E	1	3	8	−5
Butane	106-97-8	D	1	4	30	−26
2-methylpentane	107-83-5	D	2	4	18	−14
Toluene	108-88-3	D	3	4	10	−6
**Ethylacetate**	**141-78-6**	**D**	**4**	**4**	**8**	**−4**
**2-butanone**	**78-93-3**	**D**	**5**	**4**	**12**	**−8**
2-chloroacetophenone	532-27-4	E	1	4	3	+1
Tetradecane	629-59-4	D	1	5	38	−33
3,4,5-trimethyl-1-hexene	56728-10-0	D	2	5	19	−14
2-chloroacetophenone	532-27-4	D	3	5	3	+2
**Diacetone alcohol**	**123-42-2**	**D**	**4**	**5**	**7**	**−2**
**Diacetone alcohol**	**123-42-2**	**D**	**5**	**5**	**8**	**−3**
Dodecane	112-40-3	E	1	5	6	−1
Ethylacetate	141-78-6	D	1	6	10	−4
Isopropyl alcohol	67-63-0	D	2	6	9	−3
Benzaldehyde	100-52-7	D	3	6	4	+2
**Acetone**	**67-64-1**	**D**	**4**	**6**	**14**	**−8**
**Acetone**	**67-64-1**	**D**	**5**	**6**	**18**	**−12**
Ethyl octanoate	106-32-1	E	1	6	2	+4
Propanoic acid	79-09-4	D	1	7	1	+6
Ethanol	64-17-5	D	2	7	11	−4
2-ethylhexanol	104-76-7	D	3	7	7	0
**1,2-diethyl hydrazine**	**1615-80-1**	**D**	**4**	**7**	**29**	**−22**
**Ethyl lactate**	**97-64-3**	**D**	**5**	**7**	**13**	**−6**
Decane	124-18-5	E	1	7	7	0
Methyl benzoate	93-58-3	D	1	8	5	+3
Propylene glycol	57-55-6	D	2	8	20	−12
2-butanone	78-93-3	D	3	8	18	−10
**Isopropyl alcohol**	**67-63-0**	**D**	**4**	**8**	**16**	**−8**
**Hexane**	**110-54-3**	**D**	**5**	**8**	**21**	**−13**
1-undecanol	112-42-5	E	1	8	5	+3
2-chloroacetophenone	532-27-4	D	1	9	2	+7
Acetone	67-64-1	D	2	9	10	−1
Ethylacetate	141-78-6	D	3	9	13	−4
**Propylene glycol**	**57-55-6**	**D**	**4**	**9**	**30**	**−21**
**Methyl thiocyanate**	**556-64-9**	**D**	**5**	**9**	**5**	**+4**
Cyclohexane	110-82-7	E	1	9	11	−2
Isobutyrophenone	611-70-1	D	1	10	39	−29
methylhydrazine	60-34-4	D	2	10	21	−11
Isobutyraldehyde	78-84-2	D	3	10	5	+5
**2-Hydroxyethylhydrazine**	**109-84-2**	**D**	**4**	**10**	**31**	**−21**
**Isopropyl alcohol**	**67-63-0**	**D**	**5**	**10**	**20**	**−10**
Acetone	67-64-1	E	1	10	10	0

Code, see Section 1.5, corresponding to sample identification. ODT=odor detection threshold from Devos et al., [5].

**Table 8:** Comparing rank of top 10 most concentrated VOCs with the calculated OAV in all heroin samples. Bolded font signifies 1 g real heroin (sample code F1/F2). Underlined font signifies 1 g surrogate marijuana (sample code G1).

**Compound**	**CAS**	**Sample Code**		**Rank conc.**	**Rank OAV**	**Change in ranking (Rank Conc. – Rank OAV)**
**Acetic acid**	**64-19-7**	**F**	**1**	**1**	**1**	**0**
**Isobutyraldehyde**	**78-84-2**	**F**	**2**	**1**	**1**	**0**
Acetic acid	64-19-7	G	1	1	1	0
**Ethylene oxide**	**75-21-8**	**F**	**1**	**2**	**12**	**−10**
**Isobutane**	**75-28-5**	**F**	**2**	**2**	**4**	**−2**
Ethylene oxide	75-21-8	G	1	2	11	−9
**Isobutyraldehyde**	**78-84-2**	**F**	**1**	**3**	**4**	**−1**
**4-methyldecane**	**2847-72-5**	**F**	**2**	**3**	**No ODT**	
Cyclohexane	110-82-7	G	1	3	9	−6
**Isobutane**	**75-28-5**	**F**	**1**	**4**	**9**	**−5**
**2-methylpentane**	**107-83-5**	**F**	**2**	**4**	**No ODT**	
Methyl benzoate	93-58-3	G	1	4	4	0
**4-methyldecane**	**2847-72-5**	**F**	**1**	**5**	**No ODT**	
**3,4,5-trimethyl-1-hexene**	**56728-10-0**	**F**	**2**	**5**	**No ODT**	
Ethyl cyclohexane	1678-91-7	G	1	5	No ODT	
**2-methylpentane**	**107-83-5**	**F**	**1**	**6**	**No ODT**	
**Ethylenimine**	**151-56-4**	**F**	**2**	**6**	**No ODT**	
1-butanol	71-36-3	G	1	6	7	−1
**Butyric acid**	**107-92-6**	**F**	**1**	**7**	**2**	**+5**
**2,3-dimethylbutane**	**79-29-8**	**F**	**2**	**7**	**No ODT**	
2-chloroacetophenone	532-27-4	G	1	7	3	+4
**Pentanoic acid**	**109-52-4**	**F**	**1**	**8**	**3**	**+5**
**Acetone**	**67-64-1**	**F**	**2**	**8**	**8**	**0**
Benzaldehyde	100-52-7	G	1	8	6	+2
**3,4,5-trimethyl-1-hexene**	**56728-10-0**	**F**	**1**	**9**	**No ODT**	
**3-methylhexane**	**589-34-4**	**F**	**2**	**9**	**No ODT**	
p-cymene	99-87-6	G	1	9	2	+7
**Acetone**	**67-64-1**	**F**	**1**	**10**	**11**	**−1**
**Acetic acid**	**64-19-7**	**F**	**2**	**10**	**2**	**+8**
1,2,3,4-tetramethylbenzene	488-23-3	G	1	10	5	+5

Code, see Section 1.5, corresponding to sample identification.

ODT=odor detection threshold from Devos et al. [5].
